# Enterotypes of the human gut mycobiome

**DOI:** 10.1186/s40168-023-01586-y

**Published:** 2023-08-11

**Authors:** Senying Lai, Yan Yan, Yanni Pu, Shuchun Lin, Jian-Ge Qiu, Bing-Hua Jiang, Marisa Isabell Keller, Mingyu Wang, Peer Bork, Wei-Hua Chen, Yan Zheng, Xing-Ming Zhao

**Affiliations:** 1 Department of Neurology, Zhongshan Hospital and Institute of Science and Technology for Brain-Inspired Intelligence, Fudan University, Shanghai, China; 2 CAS Key Laboratory of Molecular Virology and Immunology, Chinese Academy of Sciences (CAS), Shanghai, China; 3 State Key Laboratory of Genetic Engineering, Human Phenome Institute, School of Life Sciences, Fudan University, Shanghai, China; 4 The Academy of Medical Science, Zhengzhou University, Zhengzhou, China; 5 European Molecular Biology Laboratory, Structural and Computational Biology Unit, Heidelberg, Germany; 6 Key Laboratory of Molecular Biophysics of the Ministry of Education, Hubei Key Laboratory of Bioinformatics and Molecular Imaging, Center for Artificial Intelligence Biology, Department of Bioinformatics and Systems Biology, College of Life Science and Technology, Huazhong University of Science and Technology, Wuhan, Hubei China; 7 Max Delbrück Centre for Molecular Medicine, Berlin, Germany; 8 Department of Bioinformatics, Biocenter, University of Würzburg, Würzburg, Germany; 9 College of Life Science, Henan Normal University, Xinxiang, Henan China; 10 Huzhou Central Hospital, Affiliated Central Hospital Huzhou University, Zhejiang Province, China; 11 MOE Key Laboratory of Computational Neuroscience and Brain-Inspired Intelligence, and MOE Frontiers Center for Brain Science, Fudan University, Shanghai, China; 12 State Key Laboratory of Medical Neurobiology, Institutes of Brain Science, Fudan University, Shanghai, China; 13 International Human Phenome Institutes (Shanghai), Shanghai, China

**Keywords:** Fungi, ITS, Gut mycobiome, Metagenomics, Enterotype

## Abstract

**Background:**

The fungal component of the human gut microbiome, also known as the mycobiome, plays a vital role in intestinal ecology and human health. However, the overall structure of the gut mycobiome as well as the inter-individual variations in fungal composition remains largely unknown. In this study, we collected a total of 3363 fungal sequencing samples from 16 cohorts across three continents, including 572 newly profiled samples from China.

**Results:**

We identify and characterize four mycobiome enterotypes using ITS profiling of 3363 samples from 16 cohorts. These enterotypes exhibit stability across populations and geographical locations and significant correlation with bacterial enterotypes. Particularly, we notice that fungal enterotypes have a strong age preference, where the enterotype dominated by *Candida* (i.e., Can_type enterotype) is enriched in the elderly population and confers an increased risk of multiple diseases associated with a compromised intestinal barrier. In addition, bidirectional mediation analysis reveals that the fungi-contributed aerobic respiration pathway associated with the Can_type enterotype might mediate the association between the compromised intestinal barrier and aging.

**Conclusions:**

We show that the human gut mycobiome has stable compositional patterns across individuals and significantly correlates with multiple host factors, such as diseases and host age.

Video Abstract

**Supplementary Information:**

The online version contains supplementary material available at 10.1186/s40168-023-01586-y.

## Background

The human gut microbiome, which consists of multi-kingdom microbes of prokaryotes, viruses, protists and fungi, is essential to human health [1]. Current research mainly focuses on the prokaryotic and viral components of the gut ecology [2–4]. However, the complicated associations of other types of microorganisms, particularly fungi, with human health remain largely unknown. Although the fungal community, also known as mycobiome, comprises less than 1% of the entire human gut microbiome [5], they have been shown to be involved in disease pathogenesis and to profoundly influence the host immune system [6, 7]. For example, *Candida albicans* can cause infections in immunocompromised human hosts [8], and alterations of the gut mycobiome composition have been reported in multiple human diseases [9, 10]. While fine-grained fungal taxonomic markers associated with certain phenotypes have been reported [9, 11, 12], the overall structure of the gut mycobiome and the inter-individual variation in fungal composition remain unclear.

Enterotypes, which have been proposed to summarize the human gut microbial characteristics, are effective in stratifying populations and providing a global overview of the inter-individual variations in gut microbial composition [13, 14]. Multiple studies have consistently identified bacterial enterotypes, which are independent of the distribution of the hosts’ age, geography, and gender [13–16]. Defined based on the prokaryotic compositional patterns, enterotypes could enhance understanding of human health and facilitate intervention [17]. As an integral part of the human gut multi-kingdom microbiome, fungi share microhabitats with the prokaryotic microbiome in the gut through different types of interactions, such as mutualism, commensalism, and competition [18]. Notably, several fungi-bacteria synergistic interactions within the human gut have been reported to be associated with human diseases. For instance, Hoarau et al. [19] found a positive inter-kingdom correlation between *Candida tropicalis* and two bacterial species, *Serratia macesecens* and *Escherichia coli*, in individuals with Crohn’s diseases. The physical interactions among these three species resulted in the formation of robust biofilms, which potentially cause host’s tissue damage and trigger specific immune responses [20]. Hence, the interactions between fungi and bacteria within the human gut play important roles in shaping the ecology of the intestinal microbial community [18, 21]. However, the landscape of the human gut mycobiome and whether fungal enterotype-like structures exist in the human gut are unclear.

In this study, we collected 3,363 fungal sequencing samples from 16 cohorts across Europe, North America, and Asia, including 572 newly sequenced samples from China. Four fungal enterotypes were identified independently of cohorts and geographical regions and were closely correlated with bacterial enterotypes. We noticed strong effects of host phenotypes (including age and diseases) on the fungal enterotypes. Notably, the *Candida* (Can_type) enterotype, enriched in the elderly population, showed a higher prevalence in patients with multiple diseases, even beyond the age influence, and was associated with a severe compromised intestinal barrier. Furthermore, a Can_type-enriched aerobic respiration pathway mediated the association between the compromised intestinal barrier and aging. Overall, our findings elucidated the highly structured nature of the gut mycobiome and its clinical relevance to human health.

## Results

### Landscape of human gut mycobiome composition and diversity

**Fig. 1:**
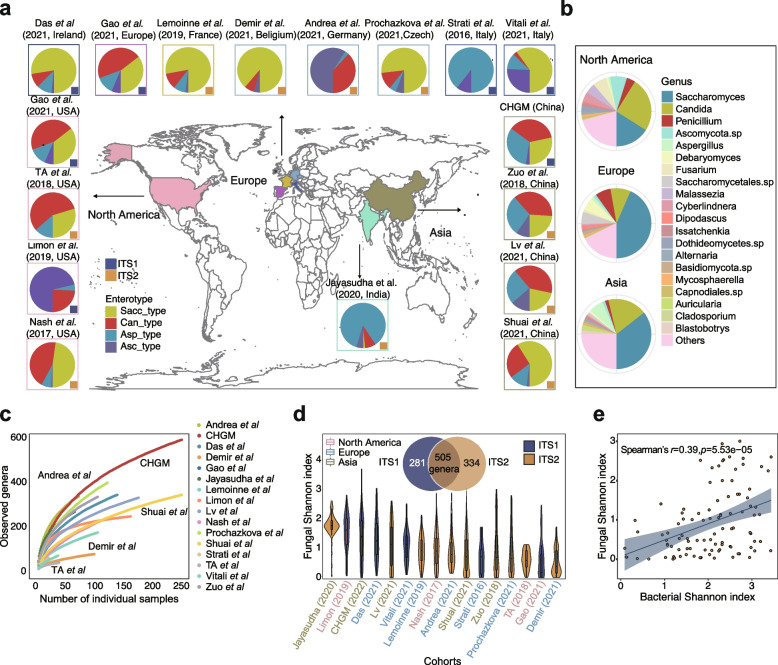
Composition and diversity of the human gut mycobiome across studies and geographic sites. **a** Geographic distribution of study populations and associated fungal enterotypes, where the datasets are sequenced with either ITS1 or ITS2 barcodes. **b** Genus-level gut mycobiome composition across the three continents (North America, Europe, and Asia). **c** Cumulative curves of the number of detected genera according to the number of sequenced samples from different study populations. **d** The distribution of fungal Shannon diversity across study populations. The Venn diagram shows the number of fungal genera detected by ITS1- and ITS2- based amplification. **e**, The correlation between the Shannon index of bacteria and that of fungi in the Zuo et al. [22] cohort, with shaded region representing 95% confidence intervals of the linear regression

To characterize the human gut fungal diversity and composition, we collected internal transcribed spacer (ITS) sequencing data from 15 published projects (Supplementary Table S1) [12, 22–30]. In addition, we recruited 572 Chinese participants (Chinese Gut Mycobiome cohort, or CHGM) aged from 17 to 89 years old and profiled their fecal mycobiome with ITS1 sequencing. In total, 3363 fecal samples with ITS1- (960 samples; hereafter referred to as ‘ITS1-combined’) or ITS2- (2403 samples; hereafter referred to as ‘ITS2-combined’) sequencing data from 16 cohorts covering three continents (Europe, North America, and Asia) were included in our study (Fig. 1a).

The combined dataset (3363 samples) contained a total of 1,120 genus-level taxonomic groups, where 354 fungi were present in at least 10 samples, and the sequencing depth of most cohorts was sufficient to capture the diversity of the gut mycobiome (Figure S2). With sample rarefaction analysis, we noticed that the number of detected genera in the Germany (Andrea et al. [28]) and Chinese (CHGM) populations dramatically increased with an increasing number of samples, and the number of fungal genera detected in our CHGM cohort far exceeded those of other cohorts (Fig. 1c, Figure S1b). However, the observed number of the fungal genera was still considerably below the estimated saturation level, even when combining all datasets (Figure S1c), suggesting a requirement for a further increase in sample size to characterize the comprehensive gut fungal diversity. At the genus level, *Saccharomyces* and *Candida* were the most abundant genera across all samples, followed by *Penicillium* and *Aspergillus* (Fig. 1b). These genera are also the most common commensal fungi in other human body sites, including skin, lung, and oral cavity [31, 32], indicating their possible well-balanced symbiotic relationship with humans.

The gut mycobiome composition and the fungal diversity varied significantly across cohorts, which may be partially attributed to biological and technical factors such as geography and sequencing methods (Fig. 1b–d, Figure S1a–d, Figure S3–4). Permutational MANOVA analysis showed that geographic location could account for approximately 3% variance of the gut mycobiome composition (*p* < 0.001, *R*^2^ = 0.03, PERMANOVA), and different abundance of specific fungal taxa across different continents was observed. Specifically, the mycobiome of European population was characterized by an expansion of *Saccharomyces* and *Penicillium* but depletion of *Candida*, while the mycobiome of populations from Asia contained a relatively higher abundance of *Candida* and lower abundance of *Saccharomyces*. The North American population harbored a relatively higher abundance of *Candida* and lower abundance of *Saccharomyces* (*p* < 0.05 with Wilcoxon rank-sum test; Figure S1a, Figure S4). Additionally, we noticed variations in fungal diversity among geographic sites (*p* < 0.05 with Kruskal–Wallis test; Figure S1d), and found that the European population displayed relatively lower fungal diversity compared with those from other continents. These results indicated that the human gut mycobiome composition is highly variable across geographical sites, similar to previous findings in the gut bacteriome [33]. Furthermore, a higher diversity was observed in ITS1-sequencing based samples compared with the ITS2-sequencing ones (Figure S1c). We found that a significant proportion of fungal taxa (~ 38%) were unique to either ITS1 or ITS2, and approximately 45% of fungal genera were detected by both methods (Fig. 1d).

The gut mycobiome, compared with the paired bacteriome, demonstrated a significantly lower Shannon diversity yet higher between-individual dissimilarity (*p* < 0.05 with Wilcoxon rank-sum test; Figure S1e). This observation is in line with previous studies showing that, in comparison with the gut bacteriome, the gut mycobiome is less diverse but more individual-specific [24, 34]. In addition, we found a positive but weak correlation between the pairwise dissimilarities of fungal and bacterial communities across studies that had matched mycobiome and bacteriome datasets (*p* < 1e − 08, Spearman’s *r*: 0.12–0.16; Figure S1f), as well as a significant positive correlation between the alpha-diversity indices of the two communities (*p* < 5.5e − 05, Spearman’s *r* = 0.39; Fig. 1e; Supplementary Table S3). These results suggest the possible mutualistic interactions between the two kingdoms within the gut.

### Enterotypes of the human gut mycobiome

**Fig. 2:**
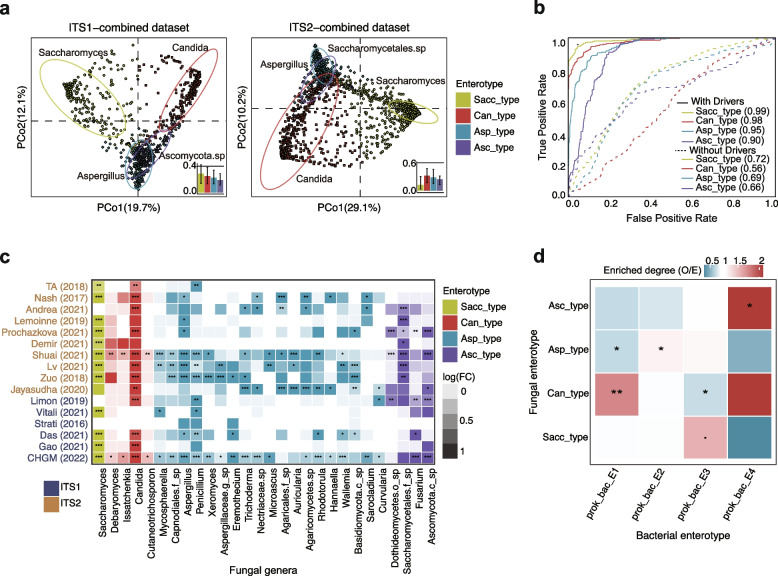
The enterotypes of the human gut mycobiome. **a** Clustering results of fungal enterotypes on ITS1 and ITS2 datasets and visualized by principal coordinate analysis (PCoA), and the most abundant genera within each enterotype is shown. The between-sample distances within each cluster compared to the median distance between clusters (black line) are shown at the bottom right of each panel. The bar height is the median distance, and the whiskers represent the 25th and 75th quantiles. **b** A four-enterotype classifier trained on the ITS2-sequencing datasets was applied to predict enterotypes in the ITS1-sequencing datasets, and the corresponding Area Under the Receiver Operating Characteristic Curve (AUC) values were presented. “Without drivers” refers to excluding the driver genera *Candida*, *Saccharomyces*, *Aspergillus*, *Saccharomycetales* sp. and *Ascomycota* sp. when training the classifiers. **c** The concordance of enterotype-associated fungal genera and enrichment trends across different cohorts, and log(FC) denotes the log-transformed fold change of the average relative abundance of the genera within respective enterotypes relative to that of others. The taxa name with a placeholder means that it could not be confidently assigned to a known taxonomic group. Asterisks represent the statistical significance of the multiple testing corrected one-sided Wilcoxon rank-sum tests (*adjusted *p* < 0.05, **adjusted *p* < 0.01, ***adjusted *p* < 0.001). **d** The correlations between fungal enterotypes and bacterial enterotypes in the CHGM cohort. The color reflects the O/E ratio (the ratio of observed count to expected count), and asterisks represent the statistical significance of Fisher’s exact test for each pair of comparison: *p < 0.05, **p < 0.01

To investigate the overall structural and compositional patterns of the human gut mycobiome, we stratified the genus-level fungal compositions of the 3363 samples into distinct groups, i.e., enterotypes (“Materials and methods” section). The clustering analysis revealed that both ITS1- and ITS2-combined datasets formed four distinct clusters (Fig. 2a, b, Figure S5a), and the clustering was also performed at different taxonomic levels with similar results, e.g., the enterotypes at the family level were highly concordant with those at the genus level (adjusted rand index > 0.5, Figure S5e; see Supplementary Note), the optimal clustering number could change at other taxonomic levels (Figure S4f). We further repeated enterotype analysis on randomly down-sampled datasets to evaluate the effect of removing samples from datasets on the overall clustering behavior. The enterotype clustering results remained unchanged even after removing half of the samples (Figure S5b, c; see Supplementary Note), further demonstrating the robustness of the fungal enterotype clusters. Three of these fungal enterotypes were found in both ITS1- and ITS2-sequencing datasets, where *Saccharomyces (*mainly species *S. cerevisiae)*, *Candida* (mainly species *C. albicans*)*,* and *Aspergillus* were the most abundant genera, respectively (Figure S6a). Therefore, we defined the *Saccharomyces-*dominated enterotype as Sacc_type, and the *Candida*- and *Aspergillus*-dominated enterotypes as Can_type and Asp_type, respectively. In addition to these three enterotypes, we also observed a fourth enterotype in both ITS1 and ITS2 (Fig. 2a). However, the fourth enterotype in ITS1 was dominated by an unclassified *Ascomycota* phylum (*Ascomycota.sp*, present in 15.1% of ITS1 samples), while in ITS2 it was driven by an unclassified *Saccharomycetales* order (*Saccharomycetales*.sp, present in 5.5% of ITS2 samples). Such a difference observed for the fourth enterotype between ITS1 and ITS2 can be attributed to different amplicon-targeted regions by ITS1 and ITS2 [35], and we found that *Ascomycota.sp* and *Saccharomycetales*.sp were enriched in ITS1- and ITS2-sequencing datasets (*p* < 0.05 with Wilcoxon rank-sum test, Figure S7), respectively. Hierarchical clustering on the combined datasets (ITS1 and ITS2) shows that these two enterotypes can be grouped together, suggesting that these two enterotypes had similar structures (Figure S5d). Thus, we defined the fourth enterotype as Asc_type hereinafter.

We further confirmed the robustness of the enterotypes by performing a cross-dataset validation analysis between the ITS1- and ITS2-combined datasets with a LASSO logistic regression model (“Materials and methods” section). In the first instance, the model’s high prediction accuracy (Fig. 2b, Figure S8) supported the fungal enterotypes’ robustness. We also obtained a good performance of cross-validation in the absence of these enterotypes’ driver genera, revealing the enterotypes’ ability to characterize the overall fungal community structure independent of the main driver genera (Fig. 2b, Figure S8). Moreover, the consistent enrichment trends of enterotype-specific fungal genera indicated that fungal enterotypes from different cohorts had a similar pattern of fungal compositions (Fig. 2c).

We then examined the geographical and ecological characterizations of the fungal enterotypes. Among the different populations, we found that the Can_type enterotype was less common in European populations (ITS1: *p* = 4.67e-14, odds ratio: 0.20; ITS2: *p* = 3.92e − 09, odds ratio: 0.44; Fisher’s exact test), while the Sacc_type enterotype was relatively rare in populations from North America (ITS1: *p* < 2.2e − 16, odds ratio: 0.04; ITS2: *p* = 1.8e − 02, odds ratio: 0.67; Fisher’s exact test). This difference might be partially attributed to the significantly decreased abundance of *Candida* in European populations and that of Saccharomyces in North American populations (*p* < 0.05 with Wilcoxon rank-sum test; Figure S1a). Furthermore, we observed that both the Sacc_type and Can_type had the lowest diversity (*p* < 0.05 with Wilcoxon rank-sum test; Figure S6b), and a strong and inverse correlation between the fungal alpha diversity indices and abundances of their respective driver genera (*p* < 2.2e − 16, Pearson’s *r* <  − 0.3).

In addition, we explored the relationship between the fungal and bacterial enterotypes using paired ITS1 for fungal profiling and metagenomics data for bacterial profiling, as both data types were available for the CHGM cohort (see “Materials and methods” section). Four bacterial enterotypes, which were identified following the same procedure as that of the fungal enterotypes with genus-level metagenomics data (Figure S9), were respectively dominated by *Bacteroides* (20.2% and 37.4% abundances in two bacterial enterotypes, annotated as prok_bac_E1 and prok_bac_E2, respectively), *Prevotella* (42.5% abundance in the prok_bac_E3 enterotype; particularly species *P. copri*) and *Enterobacteriaceae* (34.9% abundance in the prok_bac_E4). Such observations were in line with those previously reported in Asian populations [15, 36]. In addition, we observed a significant correlation between the fungal and bacterial enterotypes (*p* = 9.6e = 03, $\chi$^2^ = 21.8, chi-squared test; Fig. 2d). For example, the Can_type fungal enterotype was enriched in the prok_bac_E1 enterotype (*p* = 3.6e − 03, odds ratio: 2.41, Fisher’s exact test), and depleted in the prok_bac_E3 enterotype (*p* = 0.024, odds ratio: 0.49, Fisher’s exact test). We also observed that the Asp_type enterotype showed a trend to be enriched in the bacterial enterotypes prok_bac_E2, while the Asc_type enterotype was enriched in the prok_bac_E4 (*p* = 0.05, odds ratio = 1.56, Fisher’s exact test). Together with the consistent results from other studies (*p* = 3.4e − 07, $\chi$^2^ = 40.6, chi-squared test; Figure S10), such evidence suggested a significant correlation between fungal and bacterial communities.

### Age has a large effect on fungal enterotypes

**Fig. 3:**
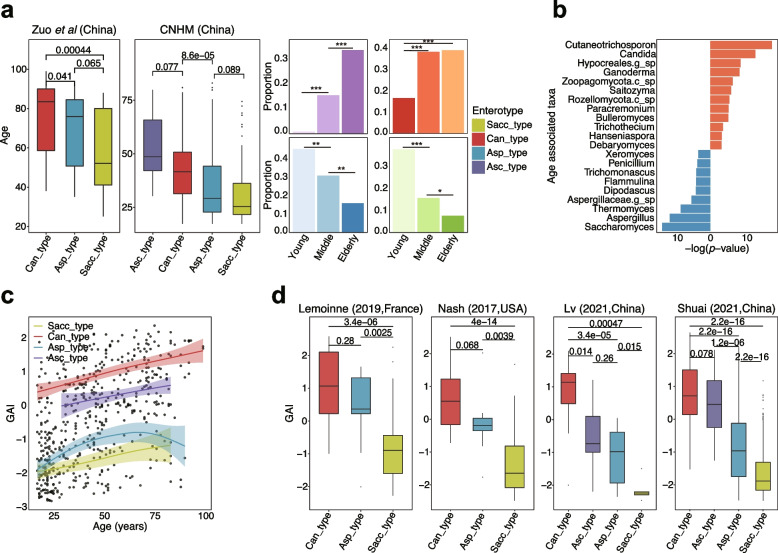
Age distribution and the gut aging indices of fungal enterotypes. **a** Age distribution of fungal enterotypes in two cohorts from China with *p* values from Wilcoxon rank-sum test *p* values shown for the age difference between enterotypes (left two panels). The right panel shows the proportion of fungal enterotypes in young (18–30 years), middle (31–60 years), and old (> –60 years) age groups from these two cohorts, respectively, with asterisks showing the statistical significance of multiple testing corrected Fisher’s exact test (*adjusted *p* < 0.05, ** adjusted* p* < 0.01, *** adjusted* p* < 0.001). **b** The age-associated fungal genera with *p* values < 0.05 determined by multivariate linear regression with adjustment of gender and cohort, where the red bar represents a positive correlation while the blue one represents a negative one. **c** The correlation between the gut aging index (GAI) and age after the LOESS smoothing for each fungal enterotype on four cohorts with available age data (CHGM cohort, Gao et al. [23], Limon et al. [12], and Zuo et al. [22]). Sacc_type: *p* = 2.1e − 03, Pearson’s *r* = 0.30; Can_type: *p* = 8.4e − 10, Pearson’s *r* = 0.45; Asp_type: *p* < 3.0e − 06, Pearson’s *r* = 0.36; Asc_type: *p* = 1.3e − 02, Pearson’s *r* = 0.27. **d** The distribution of GAI across fungal enterotypes in different cohorts. Wilcoxon rank-sum test *p* values are displayed above the boxplots

We then explored the associations between the fungal enterotypes and the hosts’ basic characteristics, including age, gender and BMI. We noticed that age could significantly explain the inter-individual variation of the human gut mycobiome and strongly affected the fungal enterotypes in three of four cohorts with available age metadata, including the CHGM cohort, Limon et al. [12], and Zuo et al. [22] (*p* < 0.05, *R*^2^ 0.04–0.15, PERMANOVA; Fig. 3a, Supplementary Table S4). The small age effect on the fungal enterotypes in the other two cohorts (Gao et al. [23] and Limon et al. [12]) was likely due to their small sample size (only 31 samples in Gao et al. [23] cohort) or the monotonous composition of fungal enterotypes (72% of samples assigned to Asc_type enterotype in Limon et al. cohort [12]). As shown in Fig. 3a, Can_type (38.8%) and Asc_type (34.0%) were significantly enriched in the elderly participants (age > 60 years), while Sacc_type (37.3%) and Asp_type (44.9%) were significantly enriched in the young participants (age < 30 years; *p* < 0.05, odds ratio > 1, Fisher’s exact test). Additionally, a significant inverse correlation between fungal Shannon diversity and chronological age was observed (*p* = 3.34e − 08, Pearson’s *r* =  − 0.19). Moreover, a multivariate linear regression analysis of 531 healthy participants from these four cohorts identified 21 age-associated fungal genera by adjusting for the potential confounding effects of gender and cohort (Fig. 3b; “Materials and methods” section). Notably, nine age-associated fungal genera were observed to have a different abundance distribution among the three fungal enterotypes (Supplementary Table S5). Among these genera, *Candida,* one driver genera of the Can_type, had a positive association with age (*p* = 4.0e − 06, $\beta$ = 4.0e − 03), while two other genera, *Saccharomyces* (*p* = 1.6e − 06, $\beta$ =  − 3.5e − 03) and *Aspergillus* (*p* = 1.3e − 05, $\beta$ =  − 1.9e − 03), showed an inverse trend. This observation was consistent with the age distribution trends of their respective fungal enterotypes (Fig. 3a). Hence, we suspected that the association of fungal enterotypes with age is at least partially driven by their respective dominant fungal genera. No significant association of fungal enterotypes with BMI or gender was found in any cohort (Supplementary Table S4).

To further demonstrate the association between the fungal enterotypes and age in other cohorts without available age metadata, we calculated a gut aging index (GAI) for each sample based on the 21 age-associated fungal genera. The GAI was defined by quantifying the balance between age-positive and age-negative associated fungal taxa similar to the idea of defining the gut microbiome health index (GMHI) [37], where higher GAI scores indicate a higher level of intestinal aging (“Materials and methods” section). According to our results, the GAI showed a strong correlation with the age of participants within each enterotype (Sacc_type: *p* = 2.1e − 03, Pearson’s *r* = 0.30; Can_type: *p* = 8.4e − 10, Pearson’s *r* = 0.45; Asp_type: *p* < 3.0e − 06, Pearson’s *r* = 0.36; Asc_type: *p* = 1.3e − 02, Pearson’s *r* = 0.27; Fig. 3c). Of note, participants of the Can_type and Asc_type enterotypes had consistently higher GAI scores throughout their lifespan, while those of the Sacc_type and Asp_type had relatively lower GAI scores (Fig. 3c). Similar results found in healthy subjects of other cohorts without available age metadata further validated the significant associations of GAI scores with fungal enterotypes (Fig. 3d). Consequently, participants of the Can_type enterotype, containing more age-positive related fungi than age-negative ones, tended to have a higher intestinal age, while the physiological condition of the Sacc_type enterotype exhibited a younger state (Fig. 3c, d). Additionally, the distribution of GAI scores in participants with different bacterial enterotypes became another piece of evidence to support correlations between fungal and bacterial enterotypes. For example, participants of the prok_E3_bac enterotype (enriched in Sacc_type) had the lowest GAI scores similar to those of the Sacc_type (*p* < 0.05 with Wilcoxon rank-sum test; Figure S11d). A correlation between the Eastern Cooperative Oncology Group (ECOG) scores, a metric used to evaluate the functional status of cancer patients, and the GAI scores was observed within the CHGM cohort (*p* = 0.04, Pearson’s *r* = 0.17; Figure S11c). Furthermore, higher GAI scores, as we observed in patients with intestinal dysbiosis compared to their paired controls, might indicate the occurrence of aging-related pathological changes in the intestine (*p* < 0.05 with Wilcoxon rank-sum test; Figure S11e; Supplementary Note).

### Functional variations across fungal enterotypes

**Fig. 4:**
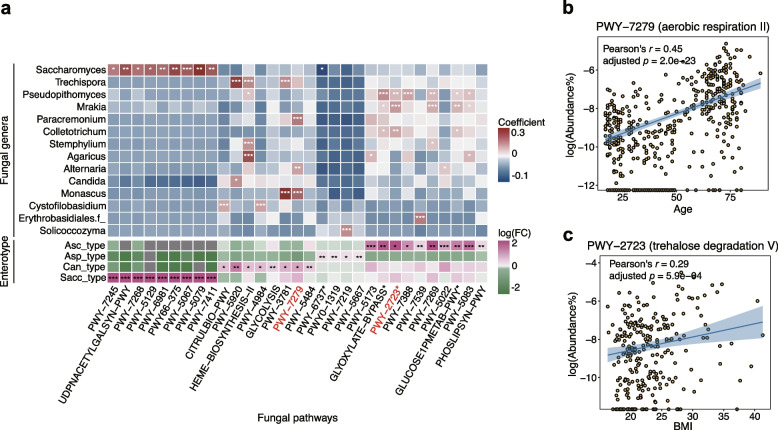
Metabolic pathways associated with fungal enterotypes. **a** The fungal pathways enriched in different fungal enterotypes (bottom) and associated fungal genera (top). Log(FC) denotes log-transformed fold change of the average relative abundance of the pathway within respective fungal enterotypes relative to that of the others. Asterisks denote the statistical significance of multiple testing corrected Pearson correlation tests (top) and multiple testing corrected Wilcoxon rank-sum tests (bottom): *adjusted *p* < 0.05, **adjusted* p* < 0.01, ***adjusted* p* < 0.001. Stars mark the metabolic pathways involved in carbohydrate degradation. **b** The relationship between the fungi-contributed pathway PWY-7279 and age. **c** The relationship between the fungi-contributed pathway PWY-2723 and BMI

To characterize the bioactive potential of the fungal enterotypes, we annotated fungi-contributed pathways based on the paired shotgun metagenomics data in the CHGM cohort (“Materials and methods” section). In total, we identified 388 biological pathways in the cohort, among which 48 were contributed by fungi alone, and 104 were contributed by both bacteria and fungi (fungi-contributed pathways hereafter). Functional richness (the observed number of fungi-contributed pathways) did not vary among fungal or bacterial enterotypes (Figure S6c). However, we identified a total of 31 fungi-contributed pathways whose distribution varied across enterotypes (adjusted *p* < 0.05 with Wilcoxon rank-sum test; Supplementary Table S6). Furthermore, the relative abundances of these pathways were also significantly correlated with those of 14 fungal genera (adjusted *p* < 0.05, Pearson’s correlation; Fig. 4a; Supplementary Table S6). An overrepresentation of pathways related to carbohydrate degradation in the Asc_type enterotype was observed, suggesting a possible increase in saccharolytic and proteolytic potential (Fig. 4a). Notably, most of the Sacc_type enriched pathways were positively associated with the relative abundance of *Saccharomyces* (Fig. 4a), which implies the essential roles of genus *Saccharomyces* in these biological pathways. Two pathways involved in heme biosynthesis (PWY-5920 and HEME-BIOSYNTHESIS-II) were enriched in the Can_type enterotype and associated with the its dominant genera, i.e., *Candida*.

We next investigated the associations between these enterotype-associated pathways with host properties. We observed a significant positive correlation between the relative abundance of the Can_type-associated pathway PWY-7279 (aerobic respiration) and subject age (adjusted *p* = 2.0e − 23, Pearson’s *r* = 0.45; Fig. 4b), consistent with the previous observation that the elderly population contained a higher abundance of pathways involved in microbial respiration [38, 39]. One of the previously detected age-positively related genera, *Paracremonium*, was also shown to be associated with aerobic respiration pathways (adjusted *p* = 2.0e − 04, Pearson’s *r* = 0.26; Fig. 3b, Fig. 4a). Moreover, we found a significant positive correlation between host BMI and PWY-2723, a trehalose degradation pathway (adjusted *p* = 5.9e − 04, Pearson’s *r* = 0.29; Fig. 4c), which might explain the slightly higher BMI levels in participants with the Asc_type enterotype (Figure S11f). The Asc_type enterotype, where PWY-2723 was enriched, had a similar enrichment of biological pathways related to energy metabolism (Fig. 4a). Thus, the functional differences observed across fungal enterotypes can partly explain the host phenotype variations among fungal enterotypes.

### Can_type enterotype is prevalent in disease populations

**Fig. 5:**
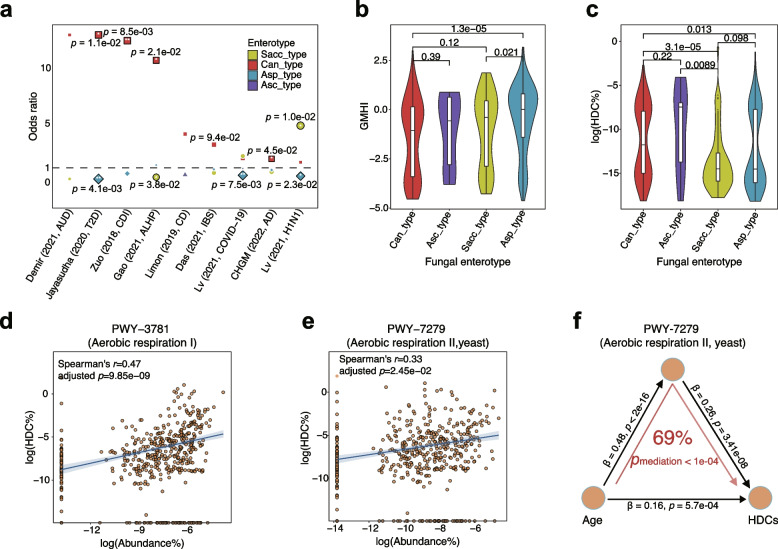
Associations between fungal enterotypes and human diseases. **a** Enrichment of the fungal enterotypes in human diseases compared to the control group after age was controlled; the odds ratios (OR) and *p* values of the Fisher’s exact test are shown. AUD: alcohol use disorder; T2D: type 2 diabetes; CDI: *clostridium difficile* infection; ALHP: alcoholic hepatitis; CD: Crohn’s disease; IBS: irritable bowel syndrome; COVID-19: coronavirus disease 2019; AD: Alzheimer’s disease. **b**,** c** Violin plots showing median and quartiles of gut microbiome health index (GMHI) (**b**) and human DNA contents (HDCs) (**c**) across fungal enterotypes in the CHGM cohort, where Wilcoxon rank-sum test *p* values are displayed above the boxplots. **d**, **e** Correlations between the HDCs (*Y*-axis) and the relative abundance of two pathways related to aerobic respiration (*X*-axis), namely PWY-7279 (**d**) and PWY-7279 (**e**). The shaded region denotes the 95% confidence interval of the linear regression. **f** Mediation linkages among the chronological age, pathway PWY-7279, and HDCs. *p*_mediation_ was estimated through a bidirectional mediation analysis with 1000 bootstraps

We further examined the clinical relevance of the fungal enterotypes by assessing their associations with human diseases. By comparing the fungal enterotype structures of healthy participants with those of patients while adjusting for age, we found that the Can_type enterotype was significantly more prevalent in patients of diseases such as type 2 diabetes, *clostridium difficile* infection, alcoholic hepatitis, and Alzheimer’s disease (Fig. 5a, *p* < 0.05, odds ratio > 1, Fisher’s exact test). Although there was no significant correlation between fungal enterotypes and other human diseases, we observed similar trends of a higher prevalence of the Can_type enterotype in the patients of these diseases (Fig. 5a, odds ratio > 1). In contrast, the other two enterotypes (i.e., the Sacc_type and the Asp_type) were mainly enriched in healthy participants (Fig. 5a; odds ratio < 1), except that the Sacc_type was enriched in two viral infectious diseases (H1N1 and COVID-19; Fig. 5a). To further quantify the disease associations across fungal enterotypes, we calculated a Gut Microbiome Health Index (GMHI) as previously described [37], and a higher GMHI value indicates a healthier status. Consistent with our expectations, the participants of the Can_type enterotype were more likely to have the lowest GMHI value (Fig. 5b), while those of the Asp_type and Sacc_type enterotypes were more likely to have higher GMHI values. Thus, in addition to its association with higher intestinal aging, the Can_type enterotype might also be related to a higher disease risk.

To explore the potential molecular mechanism contributing to the association of the Can_type enterotype with disease risk, we examined the intestinal barrier function as indicated by human DNA contents (HDCs) in the CHGM cohort (“Materials and methods” section). HDC acts as an indicator of the compromised intestinal barrier. Previous studies show a significant elevation in HDCs among patients with several intestinal diseases [40]. We found that HDCs were significantly higher in the feces of participants of the Can_type and the Asc_type enterotypes than those of the Sacc_type and the Asp_type enterotypes (*p* < 0.05 with Wilcoxon rank-sum test; Fig. 5c). This finding was consistent with the GAI scores of these enterotypes (Fig. 3c). Therefore, the compromised intestinal barrier might help to explain the increased disease risk in participants of the Can_type. In addition, we also observed significant correlations between the HDCs and the two fungi-contributed pathways involved in aerobic respiration (adjusted *p* < 0.05, spearman’s *r* > 0.3; Fig. 5d, e). These results strongly indicate significant relationships among the compromised intestinal barrier (hence the increased HDC), gut aging, and the fungal enterotypes’ distribution and bioactive potential. Furthermore, we employed bidirectional mediation analysis, a statistical technique that enables the investigation of reciprocal relationships between two variables and a mediator variable, to see whether the aerobic respiration pathway can serve as a mediator for the impact of aging on the intestinal barrier. As shown in Fig. 5f, we found that the increased age might contribute to the HDC elevation by affecting the abundance of the aerobic respiration pathway (69%, *p*_mediation_ < 1e − 04; Fig. 5f), which means the increased level of aerobic respiration significantly mediated the relationship between the age and compromised gut barrier.

## Discussion

We have uncovered noteworthy associations among age, fungal enterotypes, and disease risk. Fungal diversity decreased with increasing age, a similar trend observed for the gut prokaryotic microbiome as reported in previous studies [39, 41]. A reduction in diversity is generally indicative of intestinal dysbiosis, while gut ecosystems with high species diversity might be more resistant to external environment interferences [37, 42]. Consistent with these findings, the Can_type enterotype associated with a higher disease risk, displayed the lowest fungal diversity, rendering it more vulnerable to the external perturbations. Thus, the age-related decline in diversity might reflect a progressive loss of homeostasis in the gut ecosystem. Additionally, non-healthy participants exhibited elevated GAI scores, suggesting the potential involvement of age-related fungal genera in pathogenesis. These findings support the previous conclusion on the overlap between aging-related and disease-related deterioration in the gut microbiome [43]. The shared mycobiome alterations might be partially attributable to aging-associated disorders such as frailty and cognitive decline. In addition to the aging-associated pathological changes, the dietary habits, lifestyle, and administration of antibiotics, which can significantly affect our gut microbiome [44, 45], also vary during different stages of human life [46]. Thus, age is associated with a combination of multiple factors, which, in turn, affect fungal enterotypes. Given the occurrence of age-related changes in both the human gut mycobiome and bacteriome, we recommend combining both for future research into the underlying mechanisms of the gut microbiomes during the aging process.

The inter-kingdom interactions between the mycobiome and bacteriome are often observed in the human gut, which may mediate the observed relationship between fungal enterotypes and host physiology. For instance, the increased inflammation associated with the Candida enterotype (Can_type) could be partially mediated by mutualistic interactions between *Candida* spp and various bacterial pathogens, such as *Escherichia coli* and *Clostridium difficile*. Specifically, the virulence factors produced by *Candida* spp could enhance host susceptibility to the proliferation of these bacterial pathogens, which are known to trigger intestinal inflammation via the production of toxins [19, 47–49]. Additionally, the Sacc_type enterotype, characterized by an overabundance of *S. cerevisiae*, exhibited enhanced intestinal barrier function. *S. cerevisiae* has a protective effect on intestinal barrier integrity [50]. Although the exact mechanism of this effect is not yet fully understood, one possibility is that *S. cerevisiae* could promote the growth of several lactic acids bacteria, e.g., *Lactococcus lactis*, which in turn produce lactic acids that augment mucin production and strengthen intestinal tight junction integrity [51, 52]. Considering the bidirectional interaction between fungi and bacteria, as well as their symbiotic relationship with the human host, a more refined population stratification considering both fungal and bacterial communities might be more effective for disease diagnosis.

The functional differences arising from fungal taxonomic variations among fungal enterotypes may provide an alternative explanation for observed host phenotype variations across these enterotypes. Notably, two pathways involved in heme biosynthesis (PWY-5920 and HEME-BIOSYNTHESIS-II) enriched in the Can_type enterotype (Fig. 4a) could potentially have negative impacts on the host. Heme is a key iron source for pathogenic bacteria and has been linked to a higher risk of colorectal cancer (CRC) due to its potential to damage the intestinal mucosa [53, 54]. Interestingly, age-related changes were manifested by the increased metabolic pathways involved in microbial respiration, which were also found to be enriched in the Can_type enterotype. One possible explanation is that the higher oxygen level caused by inflammation related to aging promotes aerobic respiration in the gut microbiome [55]. Moreover, participants of the Asc_type enterotype with higher BMI levels had enriched biological pathways related to energy metabolism, which is consistent with previous findings that the microbiota of obese individuals has an increased capacity for energy harvest [56]. Therefore, the observed functional differences across fungal enterotypes are likely to have important implications for host phenotype variations.

We also noticed several limitations of our study. Firstly, the presence of the fungi detected in the stool samples does not necessarily indicate their long-term colonization in the gut as many of the detected fungi are also commonly found in the food and oral cavities. One longitudinal study of 42 individuals argued that fungi are transient in the human gut and do not colonize in the gut for long periods of time [57], but another large-scale study had contrary conclusion and identified several core fungal taxa that were stable over time [58]. To better unveil the colonization of fungi in the gut, profiling of active fungal community by ITS cDNA analysis is needed in future work. Secondly, the interactions between the bacteria and fungi were not explored here. The landscape of multi-kingdom interactions can provide insights into the mechanisms underlying the gut mycobiome structure and its association with host physiological conditions. Finally, we explored the functions of gut fungi based on the metagenomics data. However, the metagenomics data is dominated by bacteria, which leads to the underrepresentation of functional profiling of gut mycobiome. Fungi-enriched metagenomics sequencing can be helpful to infer the complete functional profiling of the mycobiome in the future.

## Conclusions

In this study, we characterized the human gut fungal community structures with a broad spectrum of ITS sequencing samples from 16 cohorts across 11 countries worldwide, including 572 newly ITS-profiled and metagenomically sequenced samples from China. We confirmed the existence of four fungal enterotypes that varied in taxonomic and functional compositions. These enterotypes showed close associations with both age and diseases, with the *Candida*-dominated enterotype being particularly enriched in the elderly population and associated with multiple human diseases accompanied by a compromised intestinal barrier. Bidirectional mediation analysis further revealed that the Can_type-associated fungi-contributed aerobic respiration pathway could mediate the association between aging and the compromised intestinal barrier. These findings reveal both the biological and clinical significance of fungal enterotypes and offer a new perspective on host-microbe interactions.

## Materials and methods

### Data collection

We downloaded ITS sequencing data of fecal samples from public databases including National Center for Biotechnology Information (NCBI) sequence read archive (SRA) and China National GeneBank database (CNGBdb). Samples with read number fewer than 10,000 were discarded. Due to the instability and large difference in the human gut mycobiome of infants, we excluded samples from infants. Metadata including demographics (e.g., age, gender, BMI, country) and human disease phenotypes were also retrieved from corresponding publications or databases. As a result, we collected a total of 2791 public samples from 11 countries covering multiple human disease phenotypes including *clostridium difficile* infection (CDI), alcohol use disorder (AUD), coronavirus disease 2019 (COVID-19), type 2 diabetes (T2D), irritable bowel syndrome (IBS), alcoholic hepatitis (ALHP), Crohn’s disease (CD), and melanoma. The details for each project including the number of samples, country, associated disease phenotype and used amplicon targets were listed in Supplementary Table S1.

We additionally collected human fecal samples from newly recruited 572 Chinese volunteers (CHGM cohort) with age ranging from 18 to 89 years old, where the fecal mycobiome was profiled with ITS1 amplification. Of these samples, 74 were collected from subjects with Alzheimer’s disease (AD) enrolled in Shanghai Sixth People’s Hospital, whereas others were obtained from healthy volunteers recruited in Wuhan, Shanghai, and Zhengzhou. Subjects who take antibiotics, antifungals or probiotics up to 1 month prior to sample collection were excluded from this study. The study protocol was approved by the Human Ethics Committee of the School of Life Science of Fudan University (No. BE1940) and the Ethics Committee of the Tongji Medical College of Huazhong University of Science. All subjects provided informed consent before participation and were asked to complete questionnaires. In total, the combined dataset consisted of 3363 samples from 16 cohorts and covered 11 countries from three continents, including Europe (615 samples), North America (344 samples) and Asia (2404 samples); among which, the fungal compositions of six and nine cohorts were determined by ITS1- (960 samples) and ITS2- (2403 samples) sequencing.

### DNA extraction from fecal samples

After sample collection, the fecal samples from the CHGM cohort were immediately stored on dry ice and transported to a refrigerator at – 80 ℃ within 5 h. Total DNA was extracted from fecal samples using semi-automated DNeasy PowerSoil HTP 96 Kit (Qiagen, 12,955–4) according to manufacturer’s instructions. The purified DNAs were quality-checked by 1% agarose gel, and DNA concentration and purity were determined with NanoDrop 2000 UV–vis spectrophotometer (Thermo Scientific, Wilmingtom, USA).

### ITS sequencing and procession

The mycobiome of CHGM cohort was profiled by the sequencing of Internal Transcribed Spacer (ITS), and the ITS1 hypervariable region was amplified with primer pairs ITS1F (5′-CTTGGTCATTTAGAGGAAGTAA-3′) and ITS2R (5′-GCTGCGTTCTTCATCGATGC-3′) [59] by an BI GeneAmp® 9700 PCR thermocycler (ABI, CA, USA). The PCR amplification was conducted as follows: initial denaturation at 95 ℃ for 3 min, followed by 27 cycles of denaturing at 95 ℃ for 30 s, annealing at 55 ℃ for 30 s, elongation at 72 ℃ for 45 s and a final extension at 72 ℃ for 10 min. The PCR mixtures (20 μL total value) contained 4 μL of 5 × FastPfu buffer, 2 μL of 2.5 mM dNTPs, 0.8 μL of each primer (5 μM concentration), 0.4 μL of FastPfu DNA Polymerase and 10 ng of template DNA. The PCR products were extracted from 2% agarose gel and purified using the AxyPrep DNA Gel Extraction Kit (Axygen Biosciences, Union City, CA, USA) according to manufacturer’s instructions, and further quantified using Quantus™ Fluorometer (Promega, USA). Purified amplicons were pooled and paired-end sequenced on Illumina MiSeq PE300 platform (Illumina, San Diego, USA) according to the standard protocols by Majorbio Bio-Pharm Technology Co. Ltd. (Shanghai, China).

The raw ITS reads were first demultiplexed, quality-filtered by fastp version 0.20.0 [60] and merged by FLASH version 1.2.7 [61] with the following criteria: (i) the 300 bp reads were truncated at any site with an average quality score < 20 over a 50-bp sliding window, and the truncated reads shorter than 50 bp were discarded; (ii) only overlapping sequences longer than 10 bp were assembled according to their overlapped sequence, and the maximum mismatch ratio of overlap region is 0.2. QIIME2 (version 2019.7) was used for the downstream analysis [62]. The quality-filtered ITS reads were then denoised and clustered into amplicon sequence variants (ASVs) using DADA2 [63], and chimeric sequences were identified and removed. Then the Naïve Bayes classifier trained on the UNITE reference database [64] was used for taxonomy assignment of individual ASVs. $\alpha$- and $\beta$-diversity analysis was conducted on samples at the sampling depth of 10,000 by utilizing the R packages “vegan” (version 2.5–7) [65] and “phyloseq” (version 1.34.0) [66]. $\alpha$-diversity was estimated by the Shannon index (evenness and richness of community within a sample), Simpson index (a qualitive measure of community diversity that accounts for both the number and the abundance of features), Faith’s phylogenetic diversity (or Faith’s PD; a qualitative measure of community diversity that incorporates both the phylogenetic relationship and abundance of the observed features) and richness (observed number of features). The fungal genera presented in less than 10 samples were excluded from downstream analysis.

### Metagenomics sequencing and processing

The fecal bacterial microbiome of CHGM cohort was profiled by whole-genome shotgun sequencing with Illumina HiSeq 2000 platform (Novogen, Beijing, China). DNA libraries were prepared as described previously [67]. The raw sequencing reads were quality-filtered using fastp version 0.20.0, followed by the use of Bowtie2 [68] to remove host-derived reads by mapping to the human reference genome (hg38). Quantitative profiling of the taxonomic composition of the microbial communities was performed via MetaPhlAn2 [69]. Profiling of microbial pathways was performed with HUMAnN2 v2.8.1 [70] by mapping reads to Uniref90 [71] and MetaCyc [72] reference databases. Both the abundance output of MetaPhlAn2 and HUMAnN2 were normalized into the relative abundance. We extracted the metabolic pathways of gut fungi for downstream analysis. The metabolic pathways or bacterial species presented in less than 10 samples were excluded from downstream analysis. To estimate the percentage of human DNA contents (HDCs) within CHGM cohort, we aligned the clean reads to the human reference genome with bowtie2, and the HDCs was calculated as the percentage of mapped reads to the total number of clean reads.

### 16S rRNA sequencing data processing

The 16S rRNA sequencing data available for four cohorts including Lemoinne et al. [27], Vitali et al. [73], Prochazkova et al. [30], and Zuo et al. [22] were downloaded from NCBI SRA. Raw 16S reads were quality filtered, clustered into ASVs and taxonomic annotated using QIIME2 (version 2019.7) as described above. The taxonomies of ASVs were annotated by using the SILVA database [74]. $\alpha$- and $\beta$-diversity analysis was conducted on samples at the sampling depth of 25,000. The bacterial genera presented in less than 10 samples were excluded from consideration.

### Fungal enterotype clustering

The fecal samples of ITS1 and ITS2 amplification were separately clustered into fungal enterotypes by using a partitioning around medoid (PAM) clustering method [75] as those previously described for bacterial enterotype discovery [13, 14]. Briefly, the samples were grouped into clusters with partitioning around medoid (PAM) based on the between-sample Bray–Curtis distance calculated at genus-level, where three other widely used distance matrices including Jaccard, Kulcxynski, and Jensen-Shannon distance (JSD) were also considered to validate the robustness of fungal enterotypes (Figure S4a). The optimal number of clusters was determined by the silhouette index. The driver genera of each enterotype was determined as the genus with the highest relative abundance in the enterotype.

We further validated the structural similarity of fungal enterotypes obtained separately from ITS1 and ITS2-combined fungal datasets. Specifically, we performed cross-dataset validation between ITS1 and ITS2 datasets with one dataset used for training a LASSO logistic regression model [76] to predict the fungal enterotype in the other dataset. To determine whether the fungal enterotypes can reflect the overall community structure and not only the difference of the driver genera, we further removed driver genera, *Candida*, *Saccharomyces*, *Aspergillus*, *Saccharomyces* sp and *Ascomycota* sp from the data and re-performed cross-validation as described above.

### Gut aging index

We calculated the gut aging index (GAI) by using the relative abundance of 21 age-associated gut fungal genera. Subjects with diseases or age below 18 years old were excluded from this analysis. To identify age-associated fungi, we adopted a multivariate linear regression analysis on 531 healthy subjects with age ranging from 18 to 90 years from four cohorts (i.e., the CHGM cohort, Gao et al. [23], Limon et al. [12], and Zuo et al. [22]) to examine the associations between age and the relative abundance of fungal genera with the adjustment of gender and cohort. The fungal genera associated with a *p* values < 0.05 in the linear regression test were considered as “age-associated”. We grouped these age-associated gut fungal genera into two sets ${M}_{P}$ and ${M}_{N}$, where ${M}_{P}$ was the set of fungal genera positively associated with age and vice versa for ${M}_{N}$. We then coupled these two fungal genera sets with a computational procedure (see below) to define a gut aging index (GAI) for a mycobiome sample. The GAI of sample *i* is defined as.

$$GAI=log10\left(\frac{{R}_{{M}_{P,i}}}{|{M}_{P}|}{\sum }_{j\in {M}_{P}}{x}_{j,i}/\frac{{R}_{{M}_{N,i}}}{|{M}_{N}|}{\sum }_{j\in {M}_{N}}{x}_{j,i}\right),$$

where ${R}_{{M}_{P,i}}$ denotes the richness of ${M}_{P}$ (or the number of present fungal genera of ${M}_{P}$ in sample *i*) in sample *i*, $|{M}_{P}|$ is the size of set ${M}_{P}$ (or the overall number of fungal genera in ${M}_{P}$), ${x}_{j,i}$ denotes the relative abundance of fungi *j* in sample *i* and the same for ${R}_{{M}_{N,i}}$ and $|{M}_{N}|$. The calculation of GAI considered both the richness and the relative abundance of age-associated gut fungal genera to quantify the balance between ${M}_{P}$ and ${M}_{N}$. Due to the difference between the set sizes of ${M}_{P}$ and ${M}_{N}$, we calculated the proportion of the present fungi of these two sets for each sample ($\frac{{R}_{{M}_{P}}}{|{M}_{P}|}$ and $\frac{{R}_{{M}_{N}}}{|{M}_{N}|}$) instead of the richness ${R}_{{M}_{P}}$ and ${R}_{{M}_{N}}$. As such, a higher GAI or GAI > 0 indicates that a more age-positive related fungal profile rather than an age-negative related fungal profile in one sample, and thus suggests a higher intestinal aging degree.

### Statistical analysis

All statistical analysis were conducted using R version 4.0.5 within RStudio and all figures were visualized by using “ggplot2” package version 3.3.5 [77]. The quantification of the variance explained by factors (e.g., continent, amplicon target) was calculated using the permutational multivariate analysis of variance (PERMANOVA, permutations = 999, distance = “bray”) as implemented by the “adonis” function in the R package “vegan”. Correlation between the $\alpha$-diversity and chronological age was assessed with Spearman’s correlation. Comparisons of enterotype characteristics (e.g., diversity), host phenotypes (e.g., BMI, age, gender, disease) and health related index (e.g., HDCs, GAI, and GMHI) across fungal enterotypes were performed using Fisher’s exact test or chi-square test for categorical variables and Wilcoxon rank-sum tests for continuous variables. The pathways enriched in each enterotype were determined by using a Wilcoxon-rank-sum test, where the other three enterotypes were combined into a single group. The bi-directional mediation analysis was performed using the “*mediate*” function within the R package “mediation” (version 4.5.0) [78] with 1000 bootstrap sampling times to infer the causal role of the aging in contributing to the compromised intestinal barrier through the fungi-contributed aerobic respiration pathway. For analysis regarding multiple comparisons, the Benjamini–Hochberg false discovery rate (adjusted *p*) [79] was employed to correct for multiple testing. The results with adjusted *p* < 0.05 were considered significant without statement specially.

### Supplementary Information

**Additional file 1:** **Figure S1.** Composition and diversity of the human gut mycobiome across studies and geographic sites. a, The distribution of four highly abundant fungal taxa across three continents. b, The distribution of the number of total reads per sample across study populations. c, Cumulative curves of the number of detected genera according to the number of sequenced samples for different amplicon targets. d, The distribution of fungal Shannon diversity across study continents. e, Comparison of the Shannon diversity (left) and Bray-Curtis pairwise dissimilarities (right) of bacteriome (16S) and mycobiome (ITS) at genus level. f, The correlation between fungal Bray-Curtis distance (FBCD) and bacterial Bray-Curtis distance (BBCD), where the Bray-Curtis distance is calculated between two samples. The shaded gray region represents 95% confidence intervals of the linear regression. In boxplots, boxes span from the first to the third quantiles and black horizontal lines represent the median, with whiskers extending 1.5 times the interquartile range (IQR). *p* values of two-sided Mann-Whitney U-test are shown. **Figure S2.** Cumulative curves of the number of detected genera (fungal richness) according to the number of sequenced reads from different study populations. **Figure S3.** Principal Coordinate Analysis (PCoA) plot of fungal community composition based on Bray-Curtis dissimilarity index. Each point represents a sample and is colored by their dataset (a), continent (b) and Phenotype (c). **Figure S4.** Composition of the human mycobiome across continents with the removal of the dataset of *Limon* (2019). a, Genus-level gut mycobiome composition across the three continents (North America, Europe, and Asia). b, The distribution of four highly abundant fungal taxa across three continents. c, The composition of fungal enterotypes across continents in ITS1- and ITS2-combined datasets, respectively. To avoid the bias introduced by the dataset of *Limon* (2019) from North America (the dataset is missing the Sacc_type enterotype), we re-examined the mycobiome composition across different continents after removing the dataset of the *Limon* (2019). **Figure S5.** The robustness of fungal enterotype clustering. a, The optimal clustering number calculated within each distance-matrix determined by Silhouette score. b, The optimal cluster number under varying sampling sizes as determined by Silhouette score for ITS- and ITS2-sequencing datasets, respectively. c, The effect of removing samples from the datasets on the overall clustering behavior for ITS1- and ITS2-sequencing datasets, respectively. We repeated 100 times with different random samples removed in each iteration. The re-clustering results indicated that the enterotypes generally clustered stably with various sample size and less than 10% of samples were wrongly categorized even when half of the samples were removed. d, Hierarchical clustering on the combined ITS1 and ITS2 datasets. e, Clustering results of fungal enterotypes on ITS1 and ITS2- combined datasets visualized by Principal coordinate analysis based on Family and Order levels. The adjusted rand index (ARI) values measuring the similarity between the enterotype clustering results at the family- or order-level against that at the genus-level are shown. e, The optimal clustering number determined by Silhouette score at different taxonomic levels. Partitioning around medoid (PAM) clustering was employed based on the between-sample Bray Curtis distance. **Figure S6.** Characteristics of fungal enterotypes. a, Abundance of the main contributors of each fungal enterotype within ITS1- (top) and ITS2-combined datasets (bottom). b, The distribution of fungal diversity across fungal enterotypes within ITS1- (top) and ITS2-combined datasets (bottom) as measured by common alpha-diversity indices. c, The distribution of functional richness across fungal enterotypes within the CHGM cohort. In boxplots, boxes span from the first to the third quantiles and black horizontal lines represent the median, with whiskers extending 1.5 times the interquartile range (IQR). *p* values of two-sided Mann-Whitney U-test are shown. **Figure S7.** The distribution of the unclassified *Ascomycota* phylum (*Ascomycota.sp*) and the unclassified *Saccharomycetales* order (*Saccharomycetales.sp*) in ITS1 and ITS2 sequencing datasets. In boxplots, boxes span from the first to the third quantiles and black horizontal lines represent the median, with whiskers extending 1.5 times the interquartile range (IQR). *p* values of two-sided Mann-Whitney U-test are shown. **Figure S8.** Robust classification of fungal enterotypes across datasets. a-c, The 5-fold cross-validation results of four-enterotype classifier on ITS1-sequencing dataset (a), ITS2-sequencing dataset (b) and ITS1- and ITS2-combined datasets (c), separately. d-e, The cross-dataset validation performance of four-enterotype classifier between ITS1 and ITS2-sequencing datasets. “Without drivers” refers to excluding the driver genera *Candida*, *Saccharomyces*, *Aspergillus*,* Saccharomycetales.sp, *and* Ascomycota.sp *when training the classifiers. “Average” refers to the micro-averaging ROC curve. **Figure S9.** Bacterial enterotype clustering results for the CHGM metagenomics dataset. a-b, The optimal clustering number calculated within each distance-matrix determined by Silhouette score (a) and CH-index (b). c, Abundance of the main contributors of each bacterial enterotype within each bacterial enterotype. **Figure S10.** The inter-kingdom interactions between bacterial and fungal communities. a, The correlations between fungal enterotypes and bacterial enterotypes in cohorts with paired 16S sequencing data. The color reflects the O/E ratio (the ratio of observed count to expected count), and asterisks represent the statistical significance of Fisher’s exact test for each pair of comparison: *p < 0.05, **p < 0.01. b, The distribution of *Bacteroides* and *Prevotella* across four fungal enterotypes. In boxplots, boxes span from the first to the third quantiles and black horizontal lines represent the median, with whiskers extending 1.5 times the interquartile range (IQR). *p* values of two-sided Mann-Whitney U-test are shown. **Figure S11.** The impacts of host phenotypes on the human gut mycobiome. a, Enterotype clustering results on randomly down-sampled datasets (sampled by age group) with 50 repetitions. The left panel shows the optimal cluster number calculated within each distance-matric using Silhouette score for each, and the inner panel of which shows the adjusted rand score (ARI) compared to the original enterotype clusters for each repetition. The right panel shows the distribution of age across re-clustered enterotypes on down-sampled datasets. b, The relationship between Shannon diversity index and age for each fungal enterotype with shaded region representing 95% confidence intervals of the linear regression. c, The correlation between gut aging index (GAI) and Eastern Cooperative Oncology Group (ECOG) score with shaded region representing 95% confidence intervals of the linear regression. d, The distribution of GAI across bacterial enterotypes (E1_bac, E2_bac, E3_bac and E4_bac). e, The distribution of gut aging index (GAI) between non-healthy (Case) and healthy (Control) subjects. f, The distribution of BMI values across fungal enterotypes. g, The distribution of Shannon diversity of the human gut mycobiome between non-healthy (Case) and healthy (Control) subjects. h, The distribution of bacterial Shannon diversity between non-healthy (AD) and healthy (Control) subjects in the CHGM cohort from China. AUD: alcohol use disorder; T2D: type 2 diabetes; CDI: clostridium difficile infection; IBS: irritable bowel syndrome; COVID-19: coronavirus disease 2019; AD: Alzheimer’s disease. In boxplots, boxes span from the first to the third quantiles and black horizontal lines represent the median, with whiskers extending 1.5 times the interquartile range (IQR). *p* values of two-sided Mann-Whitney U-test are shown. **Figure S12.** Enterotype clustering results on healthy individuals a., Clustering results of fungal enterotypes on ITS1- and ITS2-sequencing healthy datasets and visualized by principal coordinate analysis (PCoA). b., The optimal cluster number under varying sampling sizes as determined by Silhouette score for ITS1- and ITS2-sequencing healthy datasets, respectively. c., The effect of removing samples from the healthy datasets on the overall clustering behavior for ITS1- and ITS2-sequencing healthy datasets. We repeated 100 times with different random samples removed in each iteration. The re-clustering results indicated that the enterotypes generally clustered stably with various sample size and less than 10% of samples were wrongly categorized even when half of the samples were removed. **Supplementary Table S1.** Statistic summary of study populations. **Supplementary Table S2.** The effect size of different factors on fungal community using permutational MANOVA. **Supplementary Table S3.** Associations between gut fungal and bacterial alpha diversity indices, where Spearman’s coefficient and corresponded *p*-values were shown in table. Results of Vitali* et al *(2021) are not shown given its small sample size to calculate correlation. **Supplementary Table S4.** The effect size of metadata variables in human gut mycobiome variation within each cohort measured by “*envfit*” function within the R package “vegan” (*p < 0.05, **p < 0.01, ***p < 0.001). **Supplementary Table S5.** 21 age-associated fungal genera (*p*-value < 0.05). The *p*-values of association between these genera and fungal enterotype are also shown. The *p*-values are determined by the multiple linear regression with adjusted for gender and study. **Supplementary Table S6.** 31 fungal enterotype-associated metabolic pathways. The *p*-values are determined by the Wilcoxon-Rank Sum test, and the Benjamini-Hochberg false discovery rate (adjusted *p*) is employed to correct for multiple testing. Log(FC) denotes the log-transformed fold change of the pathway within respective fungal enterotype relative to other three enterotypes. Supplementary note. The robustness of fungal enterotype clustering results.

## Data Availability

The raw data of the ITS1 sequencing and metagenomic sequencing of the CHGM cohort are available in the China National Center for Bioinformation (CNCB) under accession number PRJCA010668). Other datasets used for analysis are available from the corresponding author and can be downloaded from NCBI database directly (see Supplementary Table S1). All custom code used in this work is available at the following GitHub repository: https://github.com/ZhaoXM-Lab/Mycobiome_cohort_analysis.
