# Supplementary material for: Enterotypes of the human gut mycobiome
Source: Microbiome. 2023 Aug 11;11:179. doi: 10.1186/s40168-023-01586-y (PMC10416509; doi:10.1186/s40168-023-01586-y)
Supplement: Supplementary file 2 — Additional file 1: Figure S1. Composition and diversity of the human gut mycobiome across studies and geographic sites. a, The distribution of four highly abundant fungal taxa across three continents. b, The distribution of the number of total reads per sample across study populations. c, Cumulative curves of the number of detected genera according to the number of sequenced samples for different amplicon targets. d, The distribution of fungal Shannon diversity across study continents. e, Comparison of the Shannon diversity (left) and Bray-Curtis pairwise dissimilarities (right) of bacteriome (16S) and mycobiome (ITS) at genus level. f, The correlation between fungal Bray-Curtis distance (FBCD) and bacterial Bray-Curtis distance (BBCD), where the Bray-Curtis distance is calculated between two samples. The shaded gray region represents 95% confidence intervals of the linear regression. In boxplots, boxes span from the first to the third quantiles and black horizontal lines represent the median, with whiskers extending 1.5 times the interquartile range (IQR). p values of two-sided Mann-Whitney U-test are shown. Figure S2. Cumulative curves of the number of detected genera (fungal richness) according to the number of sequenced reads from different study populations. Figure S3. Principal Coordinate Analysis (PCoA) plot of fungal community composition based on Bray-Curtis dissimilarity index. Each point represents a sample and is colored by their dataset (a), continent (b) and Phenotype (c). Figure S4. Composition of the human mycobiome across continents with the removal of the dataset of Limon (2019). a, Genus-level gut mycobiome composition across the three continents (North America, Europe, and Asia). b, The distribution of four highly abundant fungal taxa across three continents. c, The composition of fungal enterotypes across continents in ITS1- and ITS2-combined datasets, respectively. To avoid the bias introduced by the dataset of Limon (2019) from North America ( [file 40168_2023_1586_MOESM1_ESM.docx]

**Supplementary Information**

**Enterotypes of the human gut mycobiome**

Senying Lai, Yan Yan, Yanni Pu, Shuchun Lin, Jian-Ge Qiu, Bing-Hua Jiang, Marisa Keller, Mingyu Wang, Peer Bork, Wei-Hua Chen, Yan Zheng, Xing-Ming Zhao

**1 Supplementary Figures**


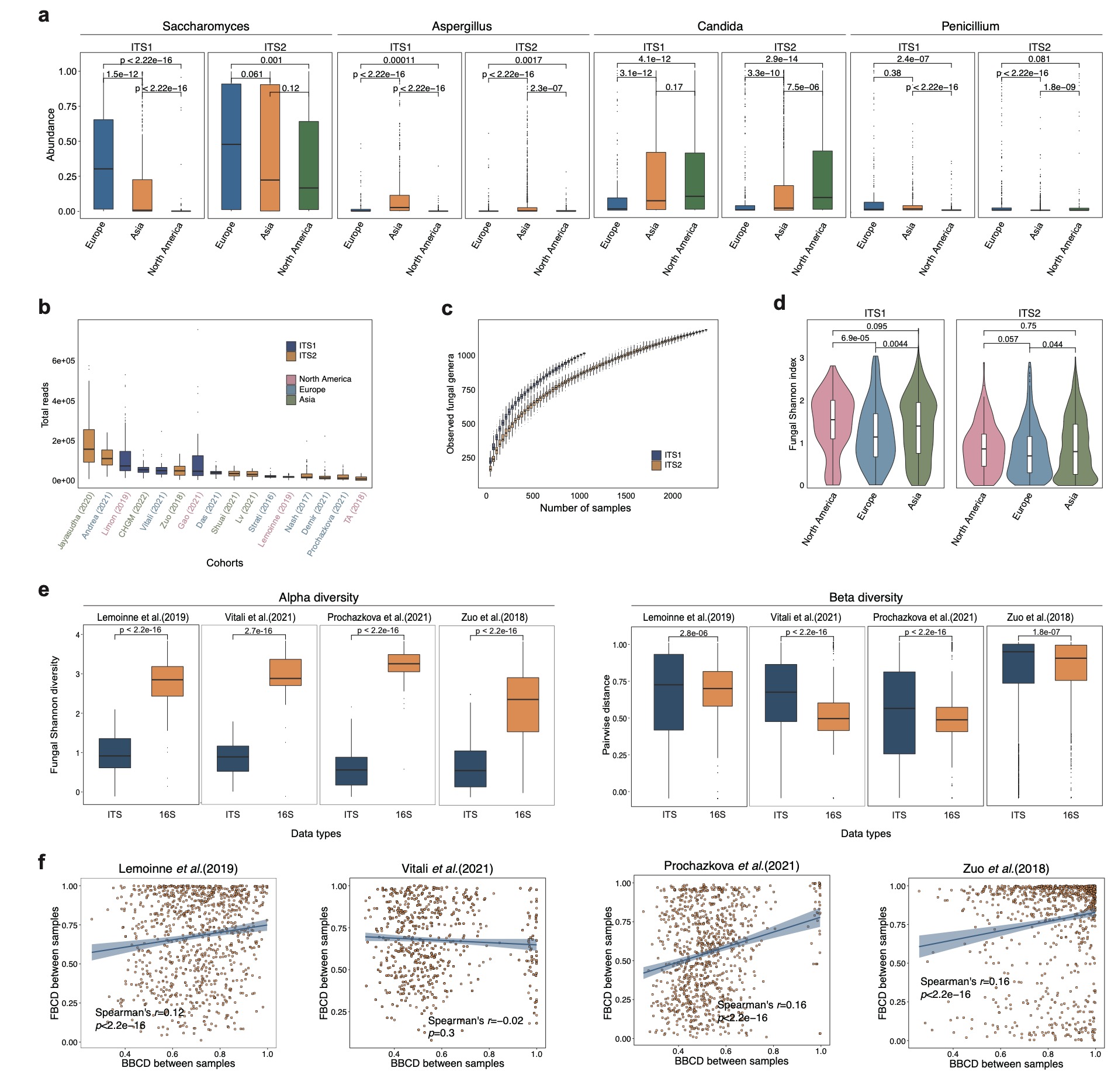


**Fig. S1**. **Composition and diversity of the human gut mycobiome across studies and geographic sites.** **a**, The distribution of four highly abundant fungal taxa across three continents. **b**, The distribution of the number of total reads per sample across study populations. **c**, Cumulative curves of the number of detected genera according to the number of sequenced samples for different amplicon targets. **d**, The distribution of fungal Shannon diversity across study continents. **e**, Comparison of the Shannon diversity (left) and Bray-Curtis pairwise dissimilarities (right) of bacteriome (16S) and mycobiome (ITS) at genus level. **f**, The correlation between fungal Bray-Curtis distance (FBCD) and bacterial Bray-Curtis distance (BBCD), where the Bray-Curtis distance is calculated between two samples. The shaded gray region represents 95% confidence intervals of the linear regression. In boxplots, boxes span from the first to the third quantiles and black horizontal lines represent the median, with whiskers extending 1.5 times the interquartile range (IQR). *p* values of two-sided Mann-Whitney U-test are shown.

**Fig. S2.** Cumulative curves of the number of detected genera (fungal richness) according to the number of sequenced reads from different study populations.

**Fig. S3**. Principal Coordinate Analysis (PCoA) plot of fungal community composition based on Bray-Curtis dissimilarity index. Each point represents a sample and is colored by their dataset (**a**), continent (**b**) and Phenotype (**c**).

**Fig. S4**. **Composition of the human mycobiome across continents with the removal of the dataset of *Limon* (2019)**. **a**, Genus-level gut mycobiome composition across the three continents (North America, Europe, and Asia). **b**, The distribution of four highly abundant fungal taxa across three continents. **c**, The composition of fungal enterotypes across continents in ITS1- and ITS2-combined datasets, respectively. To avoid the bias introduced by the dataset of *Limon* (2019) from North America (the dataset is missing the Sacc_type enterotype), we re-examined the mycobiome composition across different continents after removing the dataset of the *Limon* (2019).


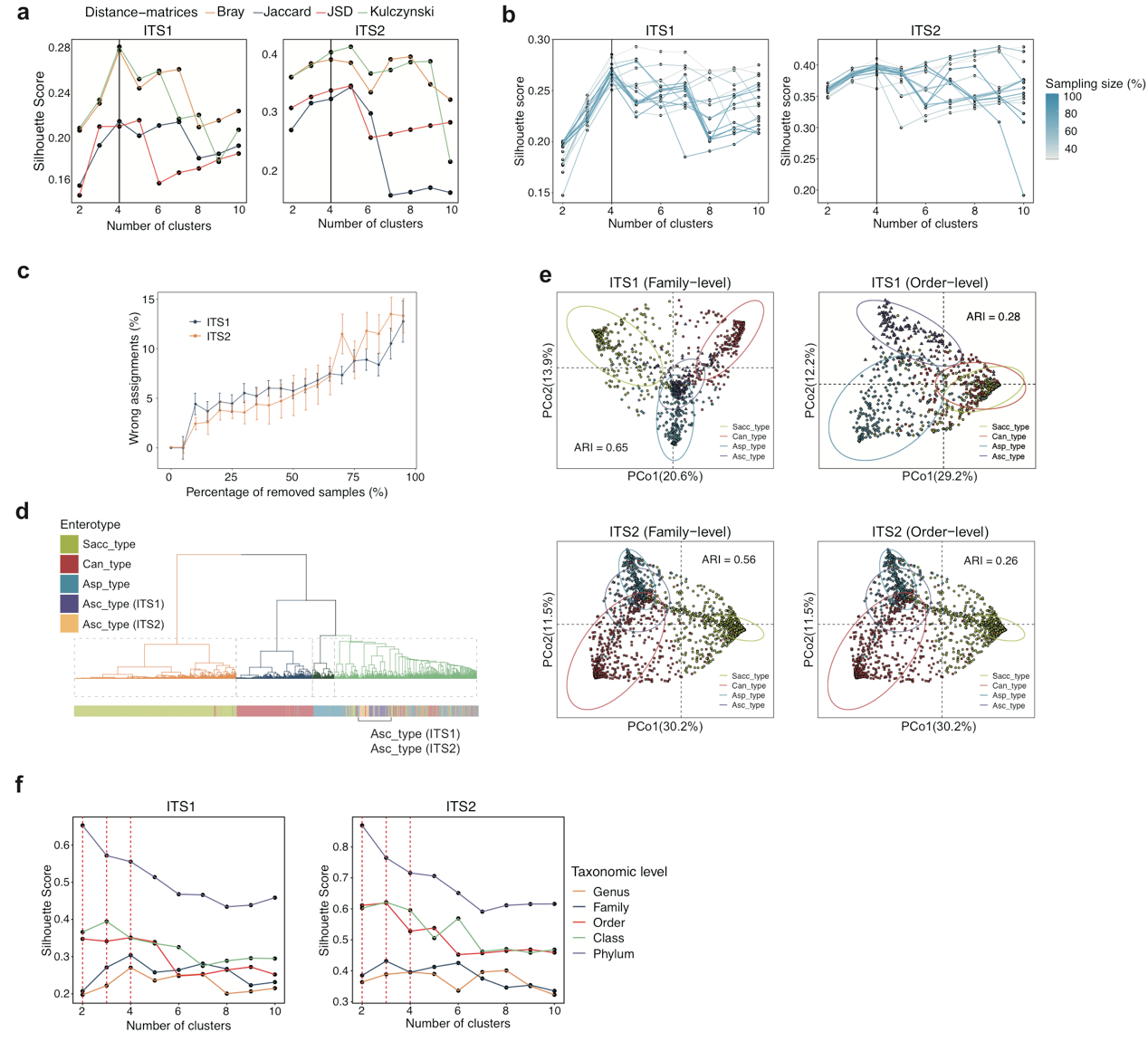


**Fig. S5**. **The robustness of fungal enterotype clustering. a**, The optimal clustering number calculated within each distance-matrix determined by Silhouette score. **b**, The optimal cluster number under varying sampling sizes as determined by Silhouette score for ITS- and ITS2-sequencing datasets, respectively. **c**, The effect of removing samples from the datasets on the overall clustering behavior for ITS1- and ITS2-sequencing datasets, respectively. We repeated 100 times with different random samples removed in each iteration. The re-clustering results indicated that the enterotypes generally clustered stably with various sample size and less than 10% of samples were wrongly categorized even when half of the samples were removed. **d**, Hierarchical clustering on the combined ITS1 and ITS2 datasets. **e**, Clustering results of fungal enterotypes on ITS1 and ITS2- combined datasets visualized by Principal coordinate analysis based on Family and Order levels. The adjusted rand index (ARI) values measuring the similarity between the enterotype clustering results at the family- or order-level against that at the genus-level are shown. **e**, The optimal clustering number determined by Silhouette score at different taxonomic levels. Partitioning around medoid (PAM) clustering was employed based on the between-sample Bray Curtis distance.

**
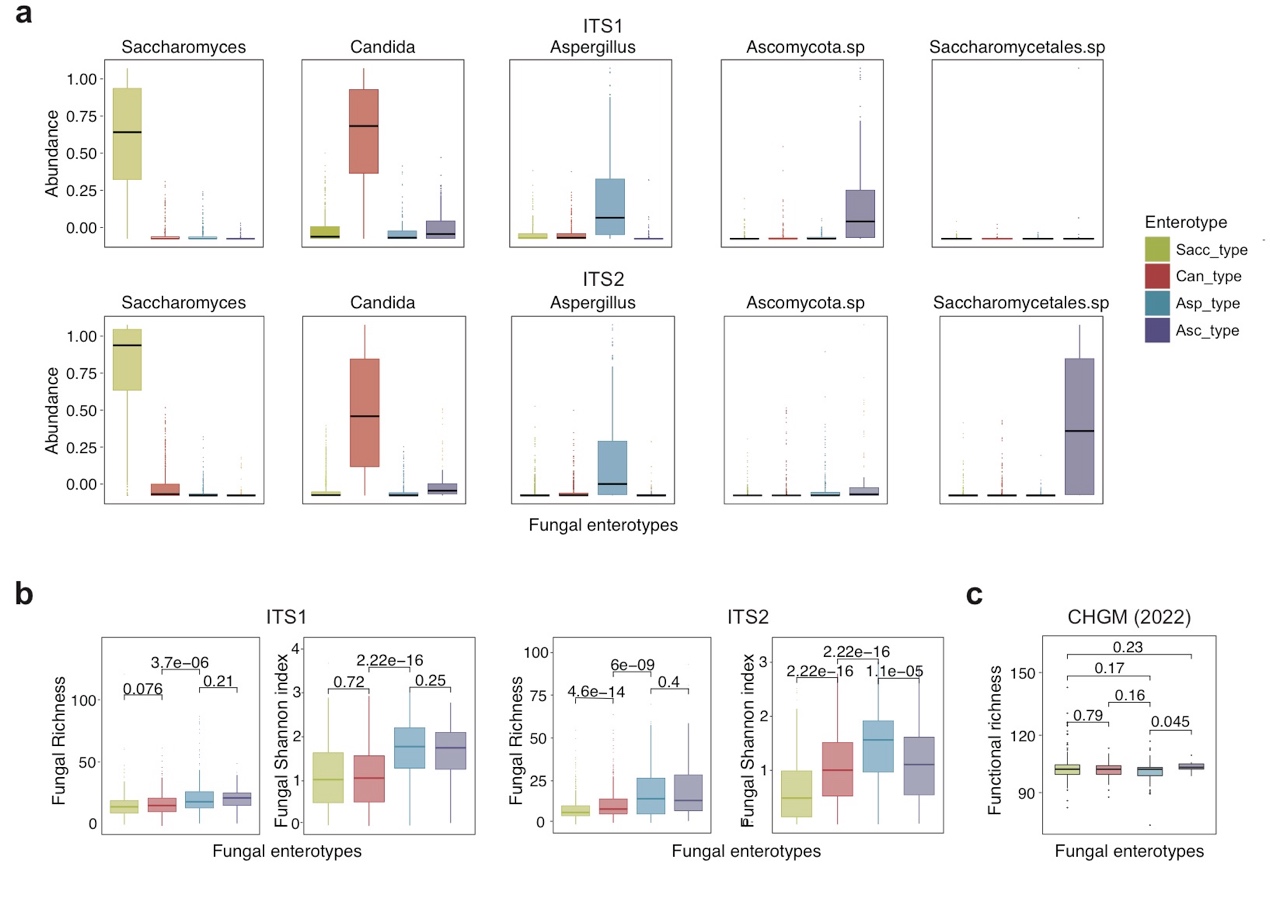
Fig. S6**. **Characteristics of fungal enterotypes. a**, Abundance of the main contributors of each fungal enterotype within ITS1- (top) and ITS2-combined datasets (bottom). **b**, The distribution of fungal diversity across fungal enterotypes within ITS1- (top) and ITS2-combined datasets (bottom) as measured by common alpha-diversity indices. **c**, The distribution of functional richness across fungal enterotypes within the CHGM cohort. In boxplots, boxes span from the first to the third quantiles and black horizontal lines represent the median, with whiskers extending 1.5 times the interquartile range (IQR). *p* values of two-sided Mann-Whitney U-test are shown.

**Fig. S7**. The distribution of the unclassified *Ascomycota* phylum (*Ascomycota.sp*) and the unclassified *Saccharomycetales* order (*Saccharomycetales.sp*) in ITS1 and ITS2 sequencing datasets. In boxplots, boxes span from the first to the third quantiles and black horizontal lines represent the median, with whiskers extending 1.5 times the interquartile range (IQR). *p* values of two-sided Mann-Whitney U-test are shown.

**Fig. S8**. **Robust classification of fungal enterotypes across datasets.** **a-c**, The 5-fold cross-validation results of four-enterotype classifier on ITS1-sequencing dataset (**a**), ITS2-sequencing dataset (**b**) and ITS1- and ITS2-combined datasets (**c**), separately. **d-e**, The cross-dataset validation performance of four-enterotype classifier between ITS1 and ITS2-sequencing datasets. “Without drivers” refers to excluding the driver genera *Candida*, *Saccharomyces*, *Aspergillus*, *Saccharomycetales.sp,* and *Ascomycota.sp* when training the classifiers. “Average” refers to the micro-averaging ROC curve.

**Fig. S9**. Bacterial enterotype clustering results for the CHGM metagenomics dataset. **a-b**, The optimal clustering number calculated within each distance-matrix determined by Silhouette score (**a**) and CH-index (**b**). **c**, Abundance of the main contributors of each bacterial enterotype within each bacterial enterotype.

**Fig. S10. The inter-kingdom interactions between bacterial and fungal communities. a**, The correlations between fungal enterotypes and bacterial enterotypes in cohorts with paired 16S sequencing data. The color reflects the O/E ratio (the ratio of observed count to expected count), and asterisks represent the statistical significance of Fisher’s exact test for each pair of comparison: **p* < 0.05, ***p* < 0.01. **b**, The distribution of *Bacteroides* and *Prevotella* across four fungal enterotypes. In boxplots, boxes span from the first to the third quantiles and black horizontal lines represent the median, with whiskers extending 1.5 times the interquartile range (IQR). *p* values of two-sided Mann-Whitney U-test are shown.


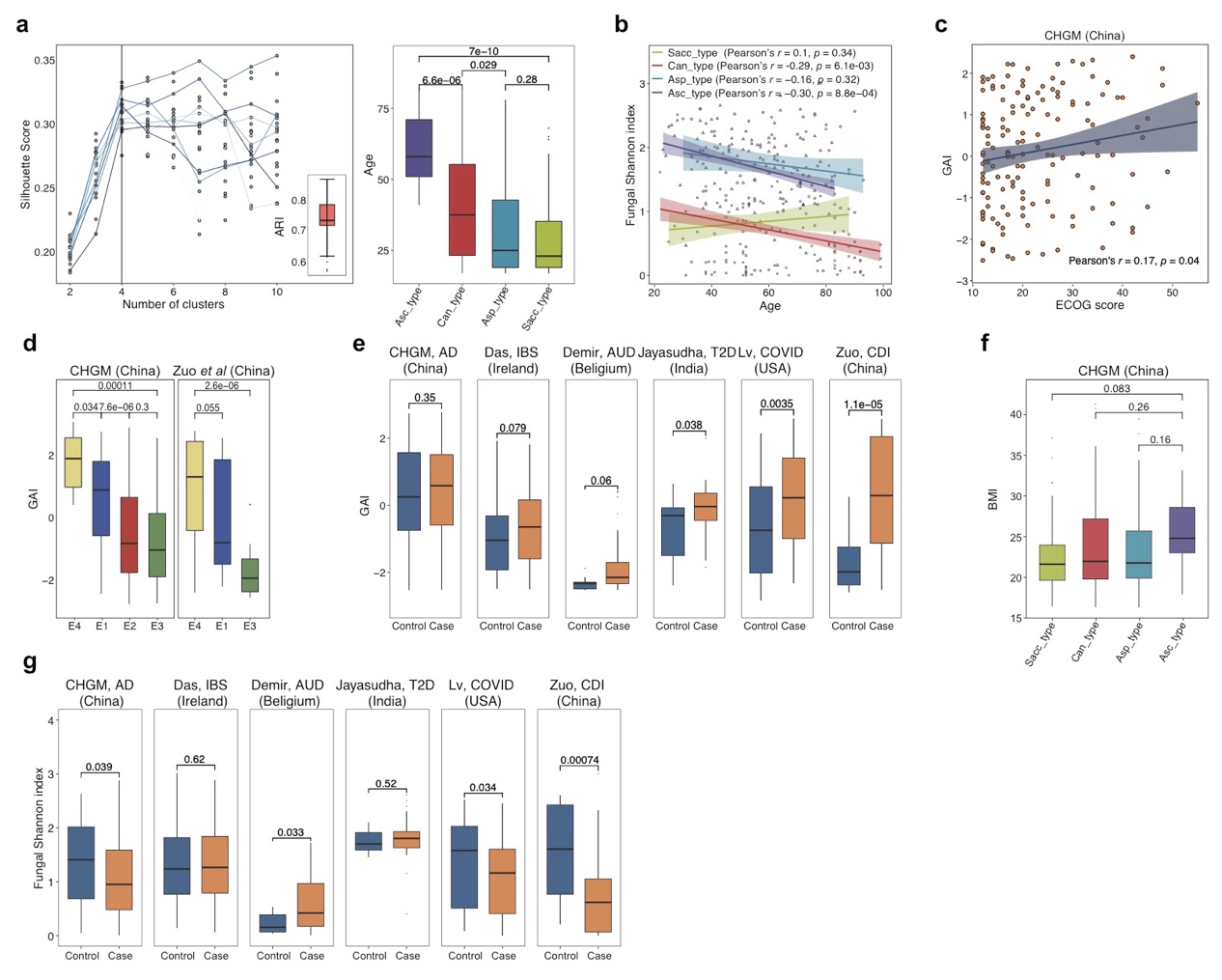


**Fig. S11.** **The impacts of host phenotypes on the human gut mycobiome.** **a**, Enterotype clustering results on randomly down-sampled datasets (sampled by age group) with 50 repetitions. The left panel shows the optimal cluster number calculated within each distance-matric using Silhouette score for each, and the inner panel of which shows the adjusted rand score (ARI) compared to the original enterotype clusters for each repetition. The right panel shows the distribution of age across re-clustered enterotypes on down-sampled datasets. **b**, The relationship between Shannon diversity index and age for each fungal enterotype with shaded region representing 95% confidence intervals of the linear regression. **c**, The correlation between gut aging index (GAI) and Eastern Cooperative Oncology Group (ECOG) score with shaded region representing 95% confidence intervals of the linear regression. **d**, The distribution of GAI across bacterial enterotypes (E1_bac, E2_bac, E3_bac and E4_bac). **e**, The distribution of gut aging index (GAI) between non-healthy (Case) and healthy (Control) subjects. **f**, The distribution of BMI values across fungal enterotypes. **g**, The distribution of Shannon diversity of the human gut mycobiome between non-healthy (Case) and healthy (Control) subjects. **h**, The distribution of bacterial Shannon diversity between non-healthy (AD) and healthy (Control) subjects in the CHGM cohort from China. AUD: alcohol use disorder; T2D: type 2 diabetes; CDI: clostridium difficile infection; IBS: irritable bowel syndrome; COVID-19: coronavirus disease 2019; AD: Alzheimer’s disease. In boxplots, boxes span from the first to the third quantiles and black horizontal lines represent the median, with whiskers extending 1.5 times the interquartile range (IQR). *p* values of two-sided Mann-Whitney U-test are shown.

**
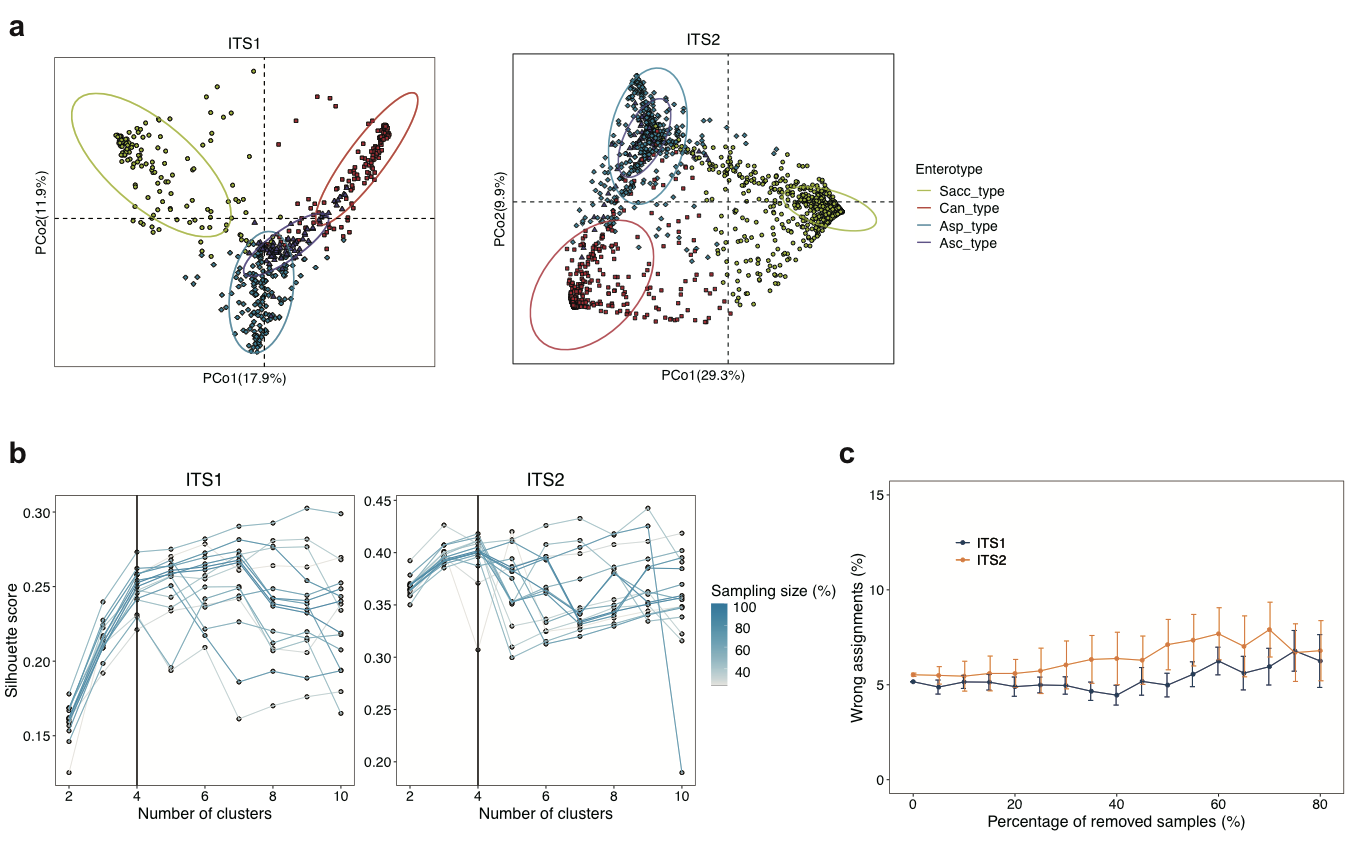
**

**Fig. S12. Enterotype clustering results on healthy individuals** **a**., Clustering results of fungal enterotypes on ITS1- and ITS2-sequencing healthy datasets and visualized by principal coordinate analysis (PCoA). **b.**, The optimal cluster number under varying sampling sizes as determined by Silhouette score for ITS1- and ITS2-sequencing healthy datasets, respectively. **c**., The effect of removing samples from the healthy datasets on the overall clustering behavior for ITS1- and ITS2-sequencing healthy datasets. We repeated 100 times with different random samples removed in each iteration. The re-clustering results indicated that the enterotypes generally clustered stably with various sample size and less than 10% of samples were wrongly categorized even when half of the samples were removed.

**2 Supplementary Tables**

**Supplementary Table 1**. Statistic summary of study populations.

| **Cohorts** | **Country** | **Associated phenotype** | **Total samples** | **Median reads per sample** | **ITS region** | **Project ID** |
| --- | --- | --- | --- | --- | --- | --- |
| Gao et al. (2021) | USA/Europe | AUD/Healthy | 31 | 46,190 | ITS1 | PRJNA517994 |
| Limon et al. (2019) | USA | CD/Healthy | 161 | 72,763 | ITS1 | PRJNA306760 |
| TA et al. (2018) | USA | Healthy | 52 | 22,953.5 | ITS2 | PRJNA379437 |
| Nash et al. (2017) | USA | Healthy | 117 | 19,333 | ITS2 | PRJNA356769 |
| Zuo et al. (2018) | China | CDI/Healthy | 104 | 54,739 | ITS2 | PRJNA419104 |
| Lv et al. (2021) | China | COVID-19/Healthy | 178 | 40,410.5 | ITS2 | PRJNA637034 |
| CHGM (2022) | China | AD/Healthy | 572 | 53,643 | ITS1 |  |
| Shuai (2021) | China |  | 1,486 | 34658.5 | ITS2 | CNP0002114 |
| Jayasudha et al. (2020) | India | T2D/Healthy | 64 | 215,337.5 | ITS2 | PRJNA662173 |
| Lemoinne et al. (2019) | France |  | 104 | 19,078 | ITS2 | PRJEB30862 |
| Andrea et al. (2021) | Germany |  | 73 | 113,304 | ITS2 | PRJNA670323 |
| Demir et al. (2021) | Belgium | AUD/Healthy | 99 | 20,034 | ITS2 | PRJNA703732 |
| Prochazkova et al.(2021) | Czech Republic |  | 126 | 38,322.5 | ITS2 | PRJEB38930 |
| Strati et al. (2016) | Italy | Healthy | 20 | 21,320.5 | ITS1 | PRJEB11827 |
| Vitali et al. (2021) | Italy | Melanoma/Healthy | 38 | 49,223 | ITS1 | PRJEB35665 |
| Das et al. (2021) | Ireland | IBS/Healthy | 138 | 39,492 | ITS1 | PRJEB42375 |

CD: Crohn’s disease; IBS: irritable bowel syndrome; CDI: clostridium difficile infection; AUD: alcohol use disorder; COVID-19: coronavirus disease 2019; AD: Alzheimer’s disease; T2D: type 2 diabetes.

**Supplementary Table 2**. The effect size of different factors on fungal community using permutational MANOVA.

| **Variable** | **R2** | ***P*-value** |
| --- | --- | --- |
| Cohort | 0.16 | *p*<0.001 |
| Enterotype | 0.38 | *p*<0.001 |
| Amplicon target | 0.027 | *p*<0.001 |
| Continent | 0.032 | *p*<0.001 |

**Supplementary Table 3**. Associations between gut fungal and bacterial alpha diversity indices, where Spearman’s coefficient and corresponded *p*-values were shown in table. Results of Vitali *et al* (2021) are not shown given its small sample size to calculate correlation.

| Lemoinne *et al.* (2019) | | | | |
| --- | --- | --- | --- | --- |
| Bacteria/Fungal | PD | Richness | Shannon | Simpson |
| PD | 0.05(*p*=0.63) | 0.06(*p*=0.52) | -0.14(*p*=0.16) | -0.14(*p*=0.17) |
| Richness | 0.04(*p*=0.67) | 0.06(*p*=0.51) | -0.13(*p*=0.20) | -0.12(*p*=0.23) |
| Shannon | 0.03(*p*=0.78) | 0.05(*p*=0.63) | -0.29(*p*=3.2e-3) | -0.25(*p*=0.01) |
| Simpson | 0.04(*p*=70) | 0.06(*p*=56) | -0.30(*p*=2.4-e3) | -0.25(*p*=9.5e-4) |
| Prochazkova *et al.* (2021) | | | | |
| Bacteria/Fungal | PD | Richness | Shannon | Simpson |
| PD | 0.16(*p*=0.08) | 0.16(*p*=0.10) | 0.11(*p*=0.25) | 0.08(*p*=0.41) |
| Richness | 0.17(*p*=0.08) | 0.15(*p*=0.11) | 0.09(*p*=0.33) | 0.06(*p*=0.50) |
| Shannon | 0.16(*p*=0.10) | 0.16(*p*=0.10) | 0.15(*p*=0.12) | 0.13(*p*=0.18) |
| Simpson | 0.08(*p*=0.43) | 0.08(*p*=0.41) | 0.09(*p*=0.34) | 0.08(*p*=0.38) |
| Zuo *et al.* (2018) | | | | |
| Bacteria/Fungal | PD | Richness | Shannon | Simpson |
| PD | 0.30(*p*=2.8e-3) | 0.27(*p*=6.0e-3) | 0.39(*p*=7.09e-05) | 0.37(*p*=1.5e-4) |
| Richness | 0.31(*p*=2.0e-3) | 0.28(*p*=4.5e-3) | 0.39(*p*=7.18e-05) | 0.37(*p*=1.6e-4) |
| Shannon | 0.37(*p*=1.5e-4) | 0.34(*p*=4.4e-4) | 0.40(*p*=5.27e-05) | 0.37(*p*=1.4e-4) |
| Simpson | 0.33(*p*=7.9e-4) | 0.31(*p*=1.7e-3) | 0.34(*p*=5.9e-4) | 0.32(*p*=1.3e-3) |

**Supplementary Table 4**. The effect size of metadata variables in human gut mycobiome variation within each cohort measured by “*envfit*” function within the R package “vegan” (**p* < 0.05, ***p* < 0.01, ****p* < 0.001).

| **Cohorts** | **BMI** | **Gender** | **Age** | **Disease** | **Enterotype** |
| --- | --- | --- | --- | --- | --- |
| Gao et al. (2021) | 0.02 | 0.01 | 0.01 | 0.18(*) | 0.88 (***) |
| Limon et al. (2019) | - | 0.01 | 0.04 (*) | 0.05 (***) | 0.34(***) |
| TA et al. (2018) | - | - | - | - | 0.72(***) |
| Nash et al. (2017) | - | - | - | - | 0.62 (***) |
| Zuo et al. (2018) | - | 0.02 | 0.15 (*) | 0.17 (***) | 0.85 (***) |
| Lv et al. (2021) | - | - | - | 0.06 (***) | 0.84 (***) |
| CHGM (2022) | 0.02 | 0.001 | 0.06 (***) | 0.05(***) | 0.84 (***) |
| Shuai (2021) |  |  |  |  |  |
| Jayasudha et al. (2020) | - | - | - | 0.09 (*) | - |
| Lemoinne et al. (2019) | - | - | - | - | 0.29 (***) |
| Andrea et al. (2021) | - | - | - | - | 0.64(***) |
| Demir et al. (2021) | - | - | - | 0.007 | 0.43(***) |
| Prochazkova et al.(2021) | - | - | - | - | 0.63(***) |
| Strati et al. (2016) | - | - | - | - | - |
| Vitali et al. (2021) | - | - | - | 0.06 | 0.29(***) |
| Das et al. (2021) | - | - | - | 0.01 | 0.57(***) |

**Supplementary Table 5**. 21 age-associated fungal genera (*p*-value < 0.05). The *p*-values of association between these genera and fungal enterotype are also shown. The *p*-values are determined by the multiple linear regression with adjusted for gender and study.

| Fungal genera | Age | | Enterotype |
| --- | --- | --- | --- |
|  | beta | *p* | *p* |
| Saccharomyces | -3.5e-03 | 1.6e-06 | 1e-3 |
| Aspergillus | -1.9e-03 | 1.3e-05 | 1e-3 |
| Thermomyces | -5.4e-04 | 2.8e-04 | 6e-3 |
| Penicillium | -5.1e-04 | 2.8e-02 | 1e-3 |
| Xeromyces | -3.9e-04 | 3.7e-02 | 3.3e-2 |
| Dipodascus | -2.9e-04 | 1.9e-02 | 3.7e-2 |
| Flammulina | -9.7e-05 | 2.0e-02 | 2.9e-2 |
| Trichomonascus | -2.5e-05 | 2.2e-02 | 5.9e-2 |
| Aspergillaceae.sp | -1.6e-05 | 5.7e-03 | 1.7e-2 |
| Bulleromyces | 4.4e-06 | 6.9e-03 | 0.9 |
| Hypocreales_fam_Incertae_sedis.sp | 7.1e-06 | 2.4e-04 | 0.82 |
| Zoopagomycota.sp | 1.1e-05 | 2.0e-03 | 0.83 |
| Trichothecium | 1.3e-05 | 3.1e-02 | 0.10 |
| Hanseniaspora | 3.2e-05 | 4.2e-02 | 0.45 |
| Rozellomycota.sp | 1.2e-04 | 5.2e-03 | 0.92 |
| Paracremonium | 1.6e-04 | 6.5e-03 | 0.57 |
| Saitozyma | 1.8e-04 | 3.2e-03 | 0.56 |
| Debaryomyces | 3.1e-04 | 4.4e-02 | 0.20 |
| Ganoderma | 5.9e-04 | 3.1e-04 | 0.27 |
| Cutaneotrichosporon | 8.5e-04 | 4.0e-08 | 0.56 |
| Candida | 4.0e-03 | 4.0e-06 | 1e-3 |

**Supplementary Table 6.** 31 fungal enterotype-associated metabolic pathways. The *p*-values are determined by the Wilcoxon-Rank Sum test, and the Benjamini-Hochberg false discovery rate (adjusted *p*) is employed to correct for multiple testing. Log(FC) denotes the log-transformed fold change of the pathway within respective fungal enterotype relative to other three enterotypes.

| **Enterotype** | **Pathway** | **Log(FC)** | **adjusted *p*** |
| --- | --- | --- | --- |
| Sacc_type | PWY-7245: superpathway NAD/NADP - NADH/NADPH interconversion (yeast) | 1.86 | 4.36e-06 |
|  | UDPNACETYLGALSYN-PWY: UDP-N-acetyl-D-glucosamine biosynthesis II | 3.32 | 4.33e-08 |
|  | PWY-7268: NAD/NADP-NADH/NADPH cytosolic interconversion (yeast) | 1.94 | 4.36e-06 |
|  | PWY-5129: sphingolipid biosynthesis (plants) | Inf | 1.33e-06 |
|  | PWY-6981: chitin biosynthesis | 3.73 | 7.44e-06 |
|  | PWY66-375: leukotriene biosynthesis | 2.96 | 3.06e-09 |
|  | PWY-5067: glycogen biosynthesis II (from UDP-D-Glucose) | 2.91 | 6.63e-06 |
|  | PWY-5079: L-phenylalanine degradation III | 3.95 | 3.06e-09 |
|  | PWY-7411: superpathway of phosphatidate biosynthesis (yeast) | 3.70 | 2.85e-06 |
| Can_type | CITRULBIO-PWY: L-citrulline biosynthesis | 0.30 | 2.45e-02 |
|  | PWY-5920: superpathway of heme biosynthesis from glycine | 0.70 | 3.92e-03 |
|  | HEME-BIOSYNTHESIS-II: heme biosynthesis I (aerobic) | 0.29 | 3.69e-02 |
|  | PWY-4984: urea cycle | 0.32 | 1.83e-02 |
|  | GLYCOLYSIS: glycolysis I (from glucose 6-phosphate) | 0.21 | 2.79e-03 |
|  | PWY-3781: aerobic respiration I (cytochrome c) | 0.38 | 2.77e-02 |
|  | PWY-7279: aerobic respiration II (cytochrome c) (yeast) | 0.45 | 2.92e-02 |
|  | PWY-5484: glycolysis II (from fructose 6-phosphate) | 0.22 | 2.79e-03 |
| Asp_type | PWY-6737: starch degradation V | 0.12 | 9.03e-03 |
|  | PWY0-1319: CDP-diacylglycerol biosynthesis II | 0.07 | 4.72e-03 |
|  | PWY-7219: adenosine ribonucleotides de novo biosynthesis | 0.11 | 1.30e-02 |
|  | PWY-5667: CDP-diacylglycerol biosynthesis I | 0.07 | 4.72e-03 |
| Asc_type | PWY-5173: superpathway of acetyl-CoA biosynthesis | 0.72 | 1.04e-04 |
|  | GLYOXYLATE-BYPASS: glyoxylate cycle | 0.94 | 2.79e-03 |
|  | PWY-2723: trehalose degradation V | 1.04 | 4.90e-02 |
|  | PWY-7388: octanoyl-[acyl-carrier protein] biosynthesis (mitochondria, yeast) | 0.59 | 4.92e-02 |
|  | PWY-7539: 6-hydroxymethyl-dihydropterin diphosphate biosynthesis III (Chlamydia) | 0.17 | 9.43e-03 |
|  | PWY-7269: NAD/NADP-NADH/NADPH mitochondrial interconversion (yeast) | 1.05 | 2.79e-03 |
|  | PWY-5022: 4-aminobutanoate degradation V | 0.46 | 4.62e-06 |
|  | GLUCOSE1PMETAB-PWY: glucose and glucose-1-phosphate degradation | 0.68 | 2.79e-03 |
|  | PWY-5083: NAD/NADH phosphorylation and dephosphorylation | 0.80 | 6.80e-04 |
|  | PHOSLIPSYN-PWY: superpathway of phospholipid biosynthesis I (bacteria) | 0.15 | 9.43e-03 |

**3 Supplementary Note**

**3.1 The robustness of fungal enterotype clustering results**

We confirmed the robustness of the enterotypes using several approaches. Firstly, we used the Silhouette score to evaluate the performance of the clustering results. Four types of between-sample distance matrices were adopted during clustering, including Jensen-Shannon distance (JSD), Bray Curtis, Jaccard and Kulcxynski distance. The optimal number of clusters was always four for four distance matrices, and the obtained clusters were concordant across four clustering results (Fig. S2a). We also quantified the effect of removing samples from datasets on the overall enterotype clustering behavior using the approach described in Arumugam *et al*[1]. Briefly, we randomly removed a certain percentage of samples from the datasets, and re-clustered. As shown in Fig. S3b-c, the re-clustering results on the down-sampled datasets indicate that the enterotypes generally clustered stably, and less than 10% of samples were wrongly categorized even when half of the samples were removed. Secondly, we trained a machine-learning model on one type of ITS-amplicon sequencing data, and tested it on the other type of ITS-amplicon sequencing data. Specifically, we trained a LASSO logistic regression model on the ITS1-combined dataset and predicted the enterotypes of ITS2-combined dataset, and *vice versa*. The high prediction accuracy (Fig. 2b, and Fig. S8) of the model indicates that the four fungal enterotypes we detect are robust across different ITS-sequencing datasets. We next measured the effect of removing driver genera on the performance of cross-validation by training models excluding the driver genera (i.e., *Candida*, *Saccharomyces*, *Aspergillus*, *Saccharomyces.sp* and *Ascomycota.sp*), we also obtained decent cross-validations, implying that the enterotypes can characterize the overall fungal community structure beyond their driver genera (Fig. 2b, and Fig. S8). Finally, we performed enterotype clustering on the fungal composition at other taxonomic levels such as family and order. The optimal clustering numbers can change at other taxonomic levels (Fig. S5f). For ITS1-sequencing datasets, the clustering numbers remained unchanged (k = 4) at the family or order levels and the optimal number decreased to three or fewer clusters at higher taxonomic levels. However, distinct fungal enterotypes also exhibited different fungal structures (Fig. S5e). For instance, PCoA plot based on family level fungal composition showed distinguishable clusters of those enterotypes, and the fungal enterotype could separately explain 38% and 40% structural variance at the family level for ITS1- and ITS2-combined datasets. Further, the fungal enterotype clusters obtained at the family level were highly concordant to those obtained at genus level (Adjusted rand index, ITS: 0.65; ITS2: 0.56). On average, 88.9% and 72.2% of samples within each genus-level enterotypes could be correctly assigned to the corresponding family-level clusters for ITS1 and ITS2 sequencing datasets, respectively. Thus, the human gut mycobiome is highly structured despite enormous inter-individual variation of fungal composition across subjects.

To remove the effect of inclusion of the diseased samples on the overall clustering of fungal enterotypes. We reperformed enterotype clustering analysis solely on healthy samples for ITS1- (562 samples) and ITS2-sequencing (1,826 samples) datasets. As shown in Fig. S12, we could still obtained similar enterotype clusters, and their robustness was also validated on randomly down-sampled datasets.

To remove the effect of unbalanced age distribution in the samples on the unsupervised clustering for enterotype discovery, we randomly sampled the same number of samples from each age group and re-did the enterotype analysis. We repeated this process 50 times and found similar fungal enterotypes and the same distribution patterns of age across enterotypes on the re-sampled datasets (Fig. S11a), further confirming the robustness of our fungal enterotypes as well as the strong correlation between age and the enterotypes.

**References**

1. Arumugam M, Raes J, Pelletier E, Le Paslier D, Yamada T, Mende DR, Fernandes GR, Tap J, Bruls T, Batto JM, et al: **Enterotypes of the human gut microbiome.** *Nature* 2011, **473:**174-180.
